# Unraveling Protein-Metabolite Interactions in Precision Nutrition: A Case Study of Blueberry-Derived Metabolites Using Advanced Computational Methods

**DOI:** 10.3390/metabo14080430

**Published:** 2024-08-03

**Authors:** Dipendra Bhandari, Kiran Kumar Adepu, Andriy Anishkin, Colin D. Kay, Erin E. Young, Kyle M. Baumbauer, Anuradha Ghosh, Sree V. Chintapalli

**Affiliations:** 1 Arkansas Children’s Nutrition Center, Little Rock, AR 72202, USA; dipendrabhandari70@gmail.com (D.B.); kkadepu@uams.edu (K.K.A.); ckay@uams.edu (C.D.K.); 2 Department of Pediatrics, University of Arkansas for Medical Sciences, Little Rock, AR 72205, USA; 3 Department of Biology, University of Maryland, College Park, MD 20742, USA; anisan@umd.edu; 4 KU Medical Center, Department of Anesthesiology, Pain and Perioperative Medicine, University of Kansas School of Medicine, Kansas City, KS 66160, USA; eyoung6@kumc.edu (E.E.Y.); kbaumbauer@kumc.edu (K.M.B.); 5 KU Medical Center, Department of Cell Biology and Physiology, University of Kansas School of Medicine, Kansas City, KS 66160, USA; 6 Department of Environmental Health, Pittsburg State University, Pittsburg, KS 66762, USA; aghosh@pittstate.edu

**Keywords:** metabolomics, in silico, target prediction, gene, protein, metabolites, computation, molecular dynamics

## Abstract

Metabolomics, the study of small-molecule metabolites within biological systems, has become a potent instrument for understanding cellular processes. Despite its profound insights into health, disease, and drug development, identifying the protein partners for metabolites, especially dietary phytochemicals, remains challenging. In the present study, we introduced an innovative in silico, structure-based target prediction approach to efficiently predict protein targets for metabolites. We analyzed 27 blood serum metabolites from nutrition intervention studies’ blueberry-rich diets, known for their health benefits, yet with elusive mechanisms of action. Our findings reveal that blueberry-derived metabolites predominantly interact with Carbonic Anhydrase (CA) family proteins, which are crucial in acid-base regulation, respiration, fluid balance, bone metabolism, neurotransmission, and specific aspects of cellular metabolism. Molecular docking showed that these metabolites bind to a common pocket on CA proteins, with binding energies ranging from −5.0 kcal/mol to −9.0 kcal/mol. Further molecular dynamics (MD) simulations confirmed the stable binding of metabolites near the Zn binding site, consistent with known compound interactions. These results highlight the potential health benefits of blueberry metabolites through interaction with CA proteins.

## 1. Introduction

Metabolomics is the systematic study of small-molecule metabolites within biological specimens. These metabolites, products of complex cellular processes and metabolism, provide insights into metabolic dynamics, health, disease, and drugs [1,2,3,4]. Metabolomics can be broadly classified into two categories, namely: (i) untargeted metabolomics, which aims to comprehensively analyze all measurable compounds within a sample, even including unknown substances; and (ii) targeted metabolomics, which focuses on quantifying targeted sets of well-defined metabolites that are characterized and quantitated relative to known chemical reference materials [5,6]. Despite its advancements, a fundamental question remains: what are the protein and gene (RNA, DNA) targets associated with these metabolites? The elucidation of this question is crucial for establishing connections between the intricate metabolic network and the pathways that govern cellular and metabolic functions, which are paramount for the pursuit of precision nutrition, medicine, and health.

Computational metabolite screening can identify novel protein targets and enhance our understanding of the intricate interplay between the molecular components that drive biological processes [7,8,9]. In silico approaches have gained prominence as valuable complementary tools to accelerate and streamline the early stages of precision health and drug discovery [10]. Utilizing these technologies empowers researchers to navigate vast databases, forecast potential targets, and prioritize candidates for subsequent experimental validation.

Previous studies have shown that dietary blueberry-derived metabolites have a profound effect on bone resorption and osteoclastogenesis in mice [11]. Metabolites such as 3-(3-hydroxyphenyl) propionic acid (3-3-PPA), hippuric acid (HA), and a mixture of seven phenolic acid metabolites identified as potentially bioactive and exhibiting the ability to stimulate osteoblast differentiation and proliferation in cell culture studies [12,13]. These compounds exert their effects on bone cells by binding to G-Protein Coupled Receptors (GPCRs), a mechanism confirmed through experimental verification involving both computational predictions and gene deletion experiments in mice [14]. In this study, we investigated 27 blueberry-derived metabolites identified in human nutrition intervention studies [15,16]. In addition to metabolites interacting with GPCRs [11,12,13,14], the present findings included several different protein targets, specifically Carbonic Anhydrase (CA) protein family members that play a pivotal role in diverse physiological processes, encompassing critical functions such as acid-base regulation, respiration, fluid balance, neurotransmission, and specific aspects of cellular metabolism.

The research presented in this study bridges metabolomics and in vitro studies, providing a preliminary model before transitioning to animal and human studies. Molecular docking analyses revealed stable binding pockets for these dietary metabolites, with binding energies comparable to those observed for experimentally validated compounds, suggesting their potential bioactivity. By identifying potential targets, such as CA family proteins, this work paves the way for further investigations and insights into potential precision nutrition, health, and therapeutic avenues.

## 2. Methods

### 2.1. Data Collection

**Figure 1:**
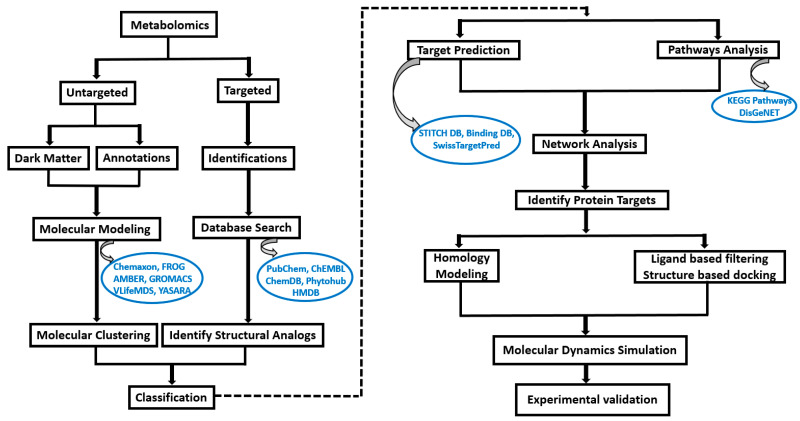
Computational workflow to determine the target protein from metabolites obtained from metabolomics. The metabolites undergo an intricate computational workflow pipeline aimed at identifying protein targets and elucidating their functional pathways. The identified target proteins and associated metabolites are then subjected to in silico methods such as molecular dynamic simulations and require further experimental validation.

In this specific study, we have demonstrated the procedure to predict various protein targets for 27 metabolites (Table 1), characterized and quantified as significantly elevated following intervention studies involving blueberries, relative to controls, utilizing targeted quantitative metabolomics approaches [15,16]. The molecular formula, structure, and SMILES for all the 27 compounds are obtained from the PubChem database (https://pubchem.ncbi.nlm.nih.gov/, accessed on 3 October 2023). The metabolites obtained from targeted metabolomics are generally commercially available compounds and also have structural and functional analog details reported in public databases, such as PubChem [17], ChEMBL [18], CheMDB [19], Phytohub v1.4 (https://phytohub.eu/, accessed on 3 October 2023) and Human Metabolome Database (HMDB) [20]. In cases where metabolites did not have reported functional analogs in the above mentioned databases, we can use various modeling tools such as Chemaxon (https://chemaxon.com/, accessed on 3 October 2023), FRee Online drug 3D conformation Generator (FROG) [21], Assisted Model Building and Energy Refinement (AMBER v24) [22], GROningen Machine for Chemical Simulations (GROMACS 2023.2) [23], V-life Molecular Design Suite (VLife MDS) and Yet Another Scientific Artificial Reality Application (YASARA) [24] to equilibrate and optimize the final structure and conformation. A detailed workflow for metabolites undergoing an intricate computational workflow pipeline aimed at identifying protein targets and elucidating their functional pathways is presented in Figure 1.

### 2.2. Molecular Clustering

All the compounds obtained after targeted metabolomics [15,16] and database search are subjected to 3D clustering and classification with Silhouette Optimized Molecular Clustering (SoMoc) and iterative Random subspace Principal Component Analysis clustering (iRaPCA) to narrow the search and cluster them in groups based on their chemical and structural similarities [25]. SoMoc is a method used to improve the clustering of molecular data by optimizing the silhouette score, which measures how similar an object is to its own cluster compared to other clusters with scores ranging from −1 to 1, with higher values indicating better clustering quality, whereas iRaPCA clustering is a technique that combines random subspace sampling with Principal Component Analysis (PCA) to improve the robustness and accuracy of clustering in high-dimensional data. Clustering small molecules, especially in large datasets, is crucial for grouping molecules with similar chemical and structural characteristics. This step helped in identifying common molecules within each cluster for all the 27 metabolites, reducing dataset complexity and making it more manageable for further analysis. Also, this approach made protein target prediction more efficient by predicting targets for representative molecules within each cluster. When handling larger datasets in the future, particularly with untargeted metabolite analysis, this simplification step will be especially beneficial in predicting targets for representative molecules rather than the entire dataset; the process becomes more focused and efficient. This study demonstrates the effectiveness of this approach using a smaller dataset, showing how clustering streamlines target prediction and enhances analysis.

### 2.3. Bioactivity Prediction

Bioactivity prediction is a valuable tool in modern science that harnesses sophisticated algorithms to anticipate potential outcomes during the testing of chemicals with previously uncharacterized biological activity. The predicted bioactivities can be diverse and include, but are not limited to, therapeutic actions, toxicological responses, enzymatic inhibition, receptor binding, or any other interaction that influences the functioning of biological systems. The bioactivity of the diet-derived blueberry metabolites in the respective clusters was predicted using Molinspiration (https://www.molinspiration.com/, accessed on 3 October 2023). Bioactivity prediction utilizes advanced computational algorithms like machine learning, artificial intelligence, and data science to forecast the potential biological effects of chemical compounds. Molinspiration scores were utilized to classify metabolites: those with scores above 0 are considered highly active, scores between 0 and −0.5 are deemed moderately active, and scores below −0.5 are categorized as inactive. This technique is used on all 27 metabolites that were previously identified as elevated, following a blueberry intervention in healthy adults [15,26,27], to identify how uncharacterized molecules might interact with biological systems, which includes cells, tissues, or whole organisms.

### 2.4. Protein Target Prediction

Protein target prediction in computational biology employs advanced algorithms and computational methods to identify and anticipate the specific proteins with which a given metabolite is likely to interact within a biological system. The selected compounds from the classification are chosen for protein target prediction. Databases such as BindingDB [28] and SwissTargetPrediction [29] are utilized for the protein target. Protein targets with a confidence score of over 90%, indicating a high likelihood of being true positives as determined through cross-validation analysis in ChEMBL [18] for considered metabolites, are selected for further analysis. Once the protein targets are identified, we performed docking and molecular dynamics simulations to access the molecular interactions, stability, binding affinity, binding orientation, and identify crucial residues involved in interaction between the targets and metabolites.

### 2.5. Metabolic Pathway and Interaction Network

Metabolic pathways are sequences of interconnected chemical reactions that transform one metabolite into another, playing a crucial role in energy production, building cellular structures, and regulating vital functions. These pathways, along with interaction networks, collectively offer a comprehensive view of the biochemical processes that govern cellular function. The potential metabolic and biological pathways affected by the metabolite and predicted target protein are identified and retrieved from the Kyoto Encyclopedia of Genes and Genomes (KEGG) database [30]. In our study, we used both the KEGG database for exploring metabolic pathways, and the STITCH database (Search Tool for InTeraction of CHemicals [31] for interaction network analysis. KEGG pathway analysis is performed for metabolites with high bioactivity score (based on the enzyme inhibition score).

### 2.6. Molecular Docking

Structure-based docking leverages advanced computational methods and three-dimensional structural information to predict and evaluate the interactions between small molecules and proteins. Molecular docking studies were performed using AutoDock Vina [32] to predict the binding modes of ligands within the target protein’s active site. The target protein structures were prepared, and the docking grid was defined with the following parameters around the Zinc (Zn) binding region, with center coordinates (x = 37.35, y = 20.63, z = −11.2) and grid box dimensions (size_x = 56, size_y = 52, size_z = 92) similar to our previous studies [11,14,33,34,35,36]. Recognizing that each PDB file has its own coordinate frame of reference, we specified parameters for each CA protein model individually. Before docking, we adjusted the center coordinates and dimensions for each CA protein model based on their specific coordinate frames. This approach ensured consistency and reliability across all CA proteins in our docking studies. The docking was configured to generate up to 10 binding modes (num_modes = 10), with an exhaustiveness value set to 100 to ensure a thorough search of the conformational space. These parameters were selected to accurately capture the potential interactions between the ligands and the target proteins, providing reliable predictions of binding affinities and orientations.

### 2.7. Molecular Dynamics (MD) Simulation

To investigate the interactions and dynamics of CAs with selected metabolites, we conducted MD simulations using NAMD 3.0 [32], similar to our previous studies [14,33,34,35,37]. The structures of the target proteins for the docking studies were retrieved from the RCSB Protein Data Bank (PDB), with corresponding PDB IDs: CA I (2CAB), CA II (1A42), CA III (1Z93), CA IV (1ZNC), CA V (1URT), CA VI (6QL2), CA VII (6SDT), CA IX (6RQN), CA XII (7PP9), CA XIII (3D0N), CA XIV (4LU3), D1 dopamine receptor (7X2F), epidermal growth factor (5WB8), estrogen receptor beta (4J24), and macrophage inhibitory factor (1CA7). The initial protein–ligand conformations were obtained from AutoDock Vina predictions (based on metabolites’ scoring in terms of binding affinity) were used as a starting structure for MD run [32]. VMD v1.9.3 [38] was utilized for the assembly of the simulation system, visualization, and analysis of the MD results, employing custom Tcl scripts. The entire system underwent energy minimization, keeping the complex fixed for 5000 steps using the conjugate gradient method to avoid conflicting contacts. This was followed by a 10 ns simulation with harmonically restrained protein backbones to equilibrate the protein side chains. Subsequently, a 100 ns unrestrained MD simulation was conducted to explore the ligand dynamics. All MD simulations were performed using the NPT ensemble with the CHARMM36 force field and the TIP3P water model [39] for both CA proteins and metabolite molecules. To ensure the electroneutrality of the system, we added necessary K+ and Cl− ions to each system individually, to an equivalent of 150 mM salt concentration. Constant pressure (1 atm) and temperature (310 K) were maintained using Langevin dynamics. Periodic boundary conditions with a flexible cell were applied, with a 12 Å cutoff for non-bonded interactions, and the particle mesh Ewald (PME) method was used for long-range electrostatic interactions with a switching distance of 10 Å. The coordinates of the system were recorded every 1 ps throughout the simulation. Based on the RMSD of the CA-metabolite complexes during the MD simulation, trajectories were extracted and analyzed.

## 3. Results and Discussion

### 3.1. Molecular Clustering

**Figure 2:**
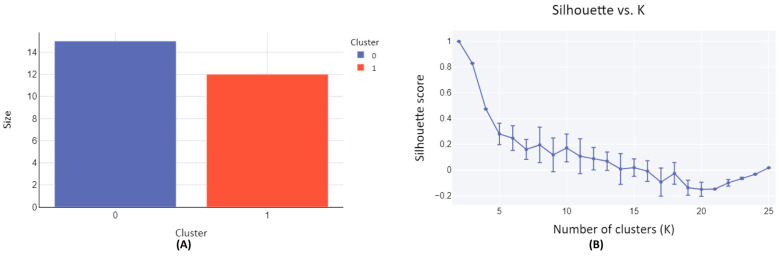
Clustering diagram of structurally similar compounds from diet-derived metabolites in human subjects following a blueberry diet intervention. (**A**) The plot depicts the relationship between cluster size and the number of clusters. Cluster-0 comprises 15 metabolite compounds, while Cluster-1 encompasses 12 metabolite compounds. (**B**) The plot illustrates the Silhouette score versus the number of clusters (K), with a Silhouette score of 0.9989 ± 0.0028 achieved for K = 2. A Silhouette score approaching 1 indicates effective clustering.

The set of 27 metabolites characterized in human subjects following a blueberry intervention, as detailed in Table 1, are clustered based on their structural similarity. The clustering process was facilitated by the employment of the SOMoC clustering algorithm using standard settings [25]. SoMoC clustering employs molecular fingerprinting to efficiently represent the structural features of molecules. These fingerprints capture the presence or absence of specific substructures within a molecule, enabling the comparison of similarities among different molecules within the metabolite group. Following fingerprinting, the Uniform Manifold Approximation and Projection (UMAP) algorithm is used to reduce the complexity of high-dimensional data while preserving its essential structure. This reduction facilitates the visualization and subsequent clustering of the data, making it easier to identify patterns and relationships among the molecules. The final step involves applying the Gaussian Mixture Model (GMM) to the UMAP-reduced data, assuming it is generated from a mixture of several Gaussian distributions, each representing a distinct cluster. As a result, the metabolites were classified into two distinct clusters, Cluster-0, and Cluster-1 (Figure 2A and Table 1). The clustering quality matrices have been evaluated by accessing a diversity of Cluster Validity Indexes (CVIs), such as Davies Bouldin (DB) score, Calinski Harabasz (CH) score, Dunn Index, and Silhouette (SIL) score, with CH and Dunn indexes’ values ranging from zero to infinity, with higher values denoting better clustering [25,40,41]. Similarly, DB score also ranges from zero to infinity but, in contrast, smaller values denote better clustering. SIL score, on the other hand, ranges from −1 to 1, with 1 representing an ideal cluster. For 100 iterations, each with 25 clusters (K), the clustering results were evaluated based on the high values of CH and Dunn scores, yielding 1009.23 ± 1.63 and 14.22 ± 0.01, respectively. These scores indicate superior clustering performance when K = 2. Furthermore, a substantial SIL score of 0.9989 ± 0.0028 was obtained for K = 2, indicating optimal clustering characterized by dense clusters well-separated from each other. This optimal configuration is visually represented in the elbow plot (Figure 2B).

Among the two clusters formed, Cluster-0 consisted of 15 metabolites, each distinguished by a singular aromatic ring and notably, a hydroxyl group. Alongside the hydroxyl group, a common feature observed in most compounds was the presence of a carbonyl group. Through the grouping of metabolites based on their structural and functional similarities, clustering has empowered this study to discern meaningful patterns and relationships within extensive and intricate datasets. The two-dimensional structure of Cluster-0 metabolites, along with their names sourced from the PubChem database, is compiled in Supplementary Figure S1. Conversely, the 12 metabolites in Cluster-1 showcased structural variations, despite all featuring an aromatic ring accompanied by a methoxy group, as illustrated in Supplementary Figure S2. This approach provided a structured basis for our proposed protein target prediction, conducted in a group-wise manner, and facilitated subsequent molecular docking analyses. The purpose of using all 27 metabolites in the subsequent analysis was to verify if the compounds within each cluster correspond to the same or similar protein targets, thereby validating our clustering approach. This verification process is crucial for ensuring the robustness of our workflow.

### 3.2. Bioactivity Scores Calculation

In the present study, we have applied the Molinspiration platform to predict the bioactivity of all the 27 metabolites previously identified as elevated following a blueberry intervention in healthy adults [15,27]. The Molinspiration tool can calculate various molecular descriptors and properties, including bioactivity predictions for small molecules. The resulting Molinspiration bioactivity scores are a prediction of drug likeliness and toxicity properties, which can be valuable for assessing the potential biological effects of small metabolites in both Cluster-0 and Cluster-1, as presented in Table 1. Organic compounds with Molinspiration scores greater than 0 are deemed highly active, those with scores between 0 and −0.5 are considered moderately active, and compounds with scores less than −0.5 are classified as inactive. The majority of the compounds in Cluster-0 have scores between 0 and −0.5, indicating moderately active protein–ligand activity. Highly active compounds are highlighted in green and moderately active compounds are highlighted in yellow (Table 1). On the other hand, most of the Cluster-1 compounds, except 4-Hydroxy-3,5-dimethoxybenzoic acid (syringic acid), 4-O-Methylgallic acid, 3-Hydroxy-4-methoxybenzoic acid, 4-hydroxy-3-methoxybenzoic acid (vanillic acid) and 4-Hydroxybenzoic acid methyl ester (methylparaben) are moderately active for enzyme inhibition activity. Compounds except 4-Hydroxybenzoic acid and 4-Hydroxybenzyl alcohol in Cluster-0 are moderately active as channel modulators. Chlorogenic acid (3-Caffeoylquinic acid) exhibits high activity in channel modulation, while all others in Cluster-0 show moderate activity. The experimental studies have shown that chlorogenic acid activates cystic fibrosis transmembrane conductance regulator (CFTR)-mediated Cl^-^ secretion in murine and human sinosasal epithelium [42]. All the metabolites in both clusters are predicted to be inactive in kinase inhibition activity, and the majority of them are also inactive as a protease inhibitor. Specifically, 3,4-Dihydroxyhydrocinnamic acid in Cluster-0 and Chlorogenic acid along with 4-Hydroxy-3,5-dimethoxycinnamic acid (sinapic acid) in Cluster-1 have a positive enzyme inhibitor score, indicative of high enzyme inhibition activity (Table 1). The enzyme inhibition activity is supported by an experimental in vitro study, where 3,4-Dihydroxyhydrocinnamic acid (caffeic acid) has shown effective antioxidant activity compared to standard antioxidant compounds such as butylated hydroxyanisole (BHA), butylated hydroxytoluene (BHT), α-tocopherol and α-torolox [43]. Accordingly, from in vitro studies on chlorogenic acid and caffeic acid, both are shown to have a higher inhibitory effect on enzymes such as α-amylase and α-glucosidase [44]. Additionally, the enzyme inhibition activity of 4-Hydroxy-3,5-dimethoxycinnamic acid (sinapic acid) against human Group IIA secreted phospholipase (sPLA2-IIA) has also been experimentally established [45].

### 3.3. Protein Target Prediction

In the present study, we used Swiss target prediction to determine the protein targets for all the metabolites. Protein targets for Cluster-0 metabolites are presented in (Table 1). CA protein family, and proteins such as aldose reductase, estrogen receptor beta, epidermal growth factor receptor, dopamine receptor, human carboxylic acid receptor 1 (HCAR1), and human carboxylic acid receptor 2 (HCAR2) are the targets for most of the Cluster-0 metabolites with a highest confidence level (90–100%). Additionally, Cluster-0 compounds had partial hits (confidence less than 90% and greater than 60%) with different proteins, such as estrogen receptor beta, tyrosine-protein kinase, aldose reductase, epidermal growth factor receptor erbB1, etc., as detailed in Supplementary Table S1. Furthermore, the CA protein family, along with various proteins such as estrogen receptor beta, epidermal growth receptor factor erbB1, and tyrosine-protein kinase proteins, also scored as the partial targets for Cluster-0 metabolites. On the other hand, very few Cluster-1 metabolites had a 90–100% confidence hit. 4-Hydroxy-3,5-dimethoxybenzoic acid (syringic acid), 4-Hydroxy-3-methoxycinnamic acid (ferulic acid), and 4-Hydroxybenzoic acid methyl ester (methylparaben) are the only Cluster-1 metabolites with 80–100% hit on CA proteins (Table 1). Cas, along with a wide range of different proteins such as aldose reductase, beta amyloid, and norepinephrine transporter, as detailed in Supplementary Table S2, are the partial hit for metabolites in Cluster-1.

### 3.4. Metabolic Pathway and Interaction Network Analysis

**Figure 3:**
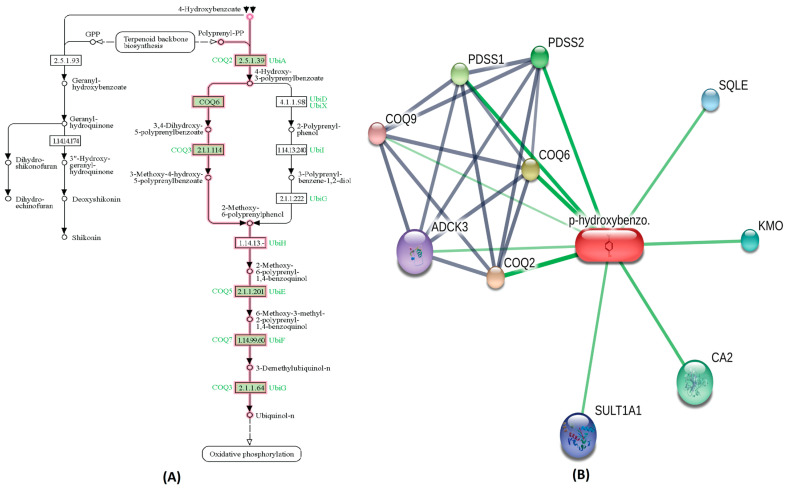
The metabolic pathway interaction network of Cluster-0 metabolite (4-hydroxybenzoic acid). (**A**) The KEGG pathway for 4-hydroxybenzoic acid involvement in oxidative phosphorylation is distinctly highlighted in a pink color. (**B**) In the STITCH interaction network for 4-hydroxybenzoic acid, Coenzyme Q2 (COQ2) and Coenzyme Q6 (COQ6), along with CA II, emerge as the nearest and most robust interaction partners, depicted by prominent green lines.

A highly bioactive metabolite from Cluster-0,3,4-Dihydroxyhydrocinnamic acid is involved in regulating the tyrosine metabolism pathway. Metabolites within Cluster-0 that exhibit moderate bioactivity played roles in specific biological pathways. For instance, 3,4,-dihydroxybenozic acid is involved in the phenylalanine, tyrosine, and tryptophan biosynthesis pathway. Another metabolite, 4-Hydroxycinnamic acid, participates in the Ubiquinone and other terpenoid-quinone biosynthesis pathway. The 2-Hydroxybenzoic acid (Salicylic acid), also present in this Cluster-0, is associated with the bile secretion pathway, while 3-Hydroxybenzoic acid contributes to the phenylalanine, tyrosine, and tryptophan biosynthesis pathway. The 4-Hydroxybenzoic acid is involved in oxidative phosphorylation pathway, as highlighted in Figure 3A. The STITCH interaction network for 4-hydroxybenzoic acid reveals its interaction with CA II, coenzyme Q2 (COQ2), coenzyme Q6 (COQ6), coenzyme Q6 (COQ6), coenzyme Q9 (COQ9), sequalene epioxidase (SQLE), Sulfotransferase1A1 (SULT1A1), aarF domain containing kinase 3 (ADCK3), Kynurenine 3-monooxygenase (KMO), prenyl (decaprenyl) diphosphate synthase, subunit 1 (PDSS1) and prenyl (decaprenyl) diphosphate synthase, subunit 2 (PDSS2), as shown in Figure 3B. Functional partners COQ2, COQ6, PDSS1, PDSS2 and CA2 have a high edge confidence score of 0.998, 0.983, 0.942, 0.927 and 0.800, respectively. Similarly, within Cluster-1, the chlorogenic acid metabolite, which exhibited a high bioactivity score as outlined in Table 1, demonstrated noteworthy functional interactions with specific proteins in the STITCH interaction network. Among its closest functional interaction partners were caspase 3 (CASP3), mitogen-activated protein kinase 8 (MAPK8), ninjurin 1 (NINJ1), DNA cytosine-5-methyl transferase 1 (DNMT1), and high mobility group box 1 (HMGB1) DNA binding proteins, each possessing high edge confidence scores of 0.817, 0.815, 0.800, 0.800, and 0.800, respectively. Conversely, the remaining compounds within Cluster-1 exhibited edge confidence scores lower than 0.800 with their interaction partners in the STITCH database. Simultaneously, the interaction networks comprising metabolites depicted complex relationships and crosstalk among common protein targets, creating a dynamic and interconnected system for identifying metabolic pathways [30].

### 3.5. Molecular Docking

**Figure 4:**
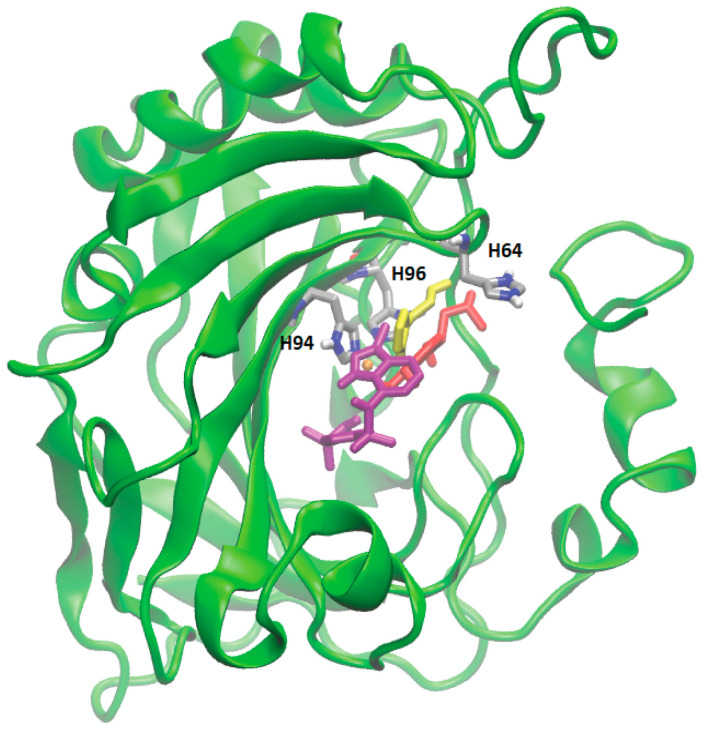
Molecular docking of four different metabolites with CA II protein. The docked state of adrenaline (in red), indisulam (in purple), histamine (in yellow), and Zn (depicted as an orange sphere) with CA II is illustrated. Adrenaline, indisulam, and histamine, recognized as experimentally proven compounds binding to CA II, are positioned within the binding site near the Zn (yellow sphere) in CA II, surrounded by histidine residues (in silver).

Proteins with a high confidence hit from protein target prediction for Cluster-0 metabolites, such as CA I to CA XIV, D(1A) dopamine receptor and epidermal growth factor receptor, along with estrogen receptor beta and macrophage migration inhibitory factor from partial hit, were selected for docking studies. The structures of the protein targets for all the tested CA family proteins are detailed in Methods, Section 2.7, with representative PDB codes provided. We used AutoDock Vina [32] for docking the Cluster-0 compounds to the target proteins. Initially, the experimentally proven compounds listed in Table 2 and known to bind to CA family proteins, obtained from Supuran et al. [46] were subjected to molecular docking to predict binding sites. The in-house docking scores for these compounds are presented in Table 2, revealing stable binding affinities with CA proteins. All the metabolites exhibit binding energies smaller than −6.0 kcal/mol, indicating a strong binding affinity towards protein targets. Figure 4 illustrates the binding site for well-known ligands of CA II, such as adrenaline, histamine, and indisulam. These compounds occupy positions near the Zn-binding site of the protein CA II, surrounded by histidine residues (HIS64, HIS94, HIS96), consistent with previous reports by Temperini et al. [47]. It is noteworthy that all the compounds listed in Table 2, bind near the Zn-binding site of CAs.

**Figure 5:**
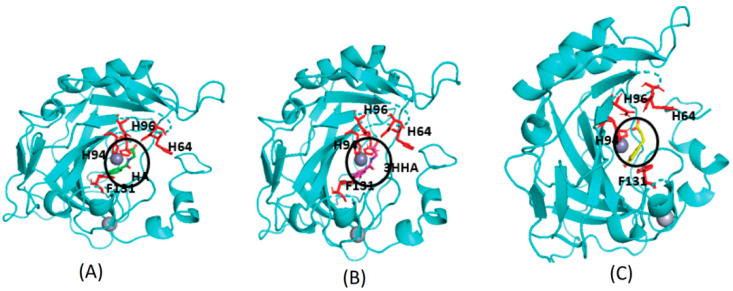
AutoDock Vina predicted binding sites for selected Cluster-0 metabolites. The docking sites for metabolites are depicted as follows: (**A**) Hippuric acid (HA) is represented in green. (**B**) 3-hydroxyhippuric acid (HHPA) is highlighted in pink. (**C**) 3,4-dihydroxycinnamic acid is shown in yellow. These metabolites are positioned near the Zn (depicted as a silver-colored sphere and highlighted within the dark circle) binding cavity of CA II, surrounded by histidine residues (HIS64, HIS94, HIS96, and PHE131). Notably, these metabolites share the identical binding site with experimentally bound compounds.

**Figure 6:**
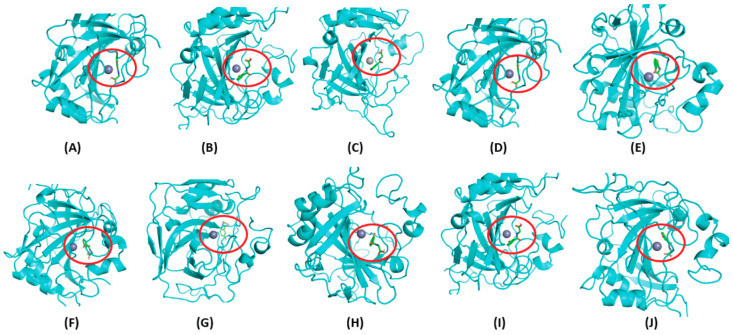
Predicted binding site of a single metabolite with all the CA family proteins. The docked state of 3-hydroxyhippuric acid, represented in green and depicted in licorice form, is illustrated near the Zn (depicted as a silver-colored sphere) binding site across various CA family proteins. The binding site of 3-hydroxyhippuric acid is consistently highlighted within the red circle for clarity. (**A**) CA II, (**B**) CA III, (**C**) CA IV, (**D**) CA V, (**E**) CA VI, (**F**) CA VII, (**G**) CA IX, (**H**) CA XII, (**I**) CA XIII, (**J**) CA XIV. Notably, 3-hydroxyhippuric acid occupies a similar binding site across all CA family proteins.

The clustering of experimentally proven compounds listed in Table 2, alongside all 27 metabolites, revealed a predominant clustering pattern with Cluster-0 metabolites. Furthermore, a visual examination comparing these compounds to Cluster-0 metabolites indicated a notable similarity in their parent backbone structures, with minimal variations observed in the side chain groups (Supplementary Figures S1 and S4). This clustering pattern suggests a shared structural resemblance between the known compounds and specific metabolites within Cluster-0. Utilizing the same binding site as the known compounds, Cluster-0 metabolites were then docked to all the CA family proteins, starting from CA I–CA XIV. All Cluster-0 metabolites occupied the binding region as known compounds near the Zn binding site of CAs. Figure 5 shows the binding site for the metabolites hippuric acid, 3-hydroxyhippuric acid and 3,4-dihydroxycinnamic acid, near the Zn binding cavity surrounded by histidine (HIS64, HIS94, HIS96 and PHE131) in CA II. This result is consistent with the X-ray crystal structures reported for CA II with pyridine-3-carboxylic acid (nicotinic acid or niacin) and 4-Hydroxy-3-methoxycinnamic acid (ferulic acid) [48]. In the context of the CA family, where it was observed that all Cluster-0 metabolites exhibited a binding site near the Zn-binding cavity. Figure 6 shows a representative metabolite from Cluster-0,3-hydroxyhippuric acid docked to Zn binding site of all CA family proteins, starting from CA I–CA XIV. These docking results have also shown that all the Cluster-0 metabolites have stable binding energies with the CA family proteins (Table 3). Additionally, other metabolites such as 3-Hydroxyhippuric acid, 4-Hydroxycinnamic acid and 3,4-Dihydroxyhydrocinnamic acid (caffeic acid) have stronger binding energies of −7.5 kcal/mol, −7.2 kcal/mol and −7.0 kcal/mol, respectively, with CA IX. However, some metabolites have relatively weaker binding, such as 2,4,6-trihydroxybenzaldehyde with CA XIII (−4.9 kcal/mol) and 4-Hydroxybenzyl alcohol with CA XII (−4.9 kcal/mol). The remaining metabolites possessed binding energy ranging from −5.0 kcal/mol to −7.5 kcal/mol, suggesting stronger binding.

In addition to the CA proteins, Cluster-0 metabolites were docked with other predicted protein targets with a high confidence hit, such as D(1A) dopamine receptor and epidermal growth factor receptor, along with estrogen receptor beta and macrophage migration inhibitory factor as partial hit targets. These metabolites have shown stable binding energy with these protein targets (Supplementary Table S3). Metabolites such as 3-Hydroxyhippuric acid have a stronger binding energy (−7.2 kcal/mol) with estrogen receptor beta, while compounds such as 4-hydroxybenzyl alcohol have a weaker binding energy (−4.7 kcal/mol) with epidermal growth factor receptor protein. Supplementary Figure S3 shows the docked state of some metabolites, such as 3-hydroxyhippuric acid with estrogen receptor beta and macrophage inhibitor factor, 3,4-dihydroxycinnamic acid with epidermal growth factor, and hippuric acid with dopamine receptor protein.

### 3.6. Molecular Dynamics Simulations

**Figure 7:**
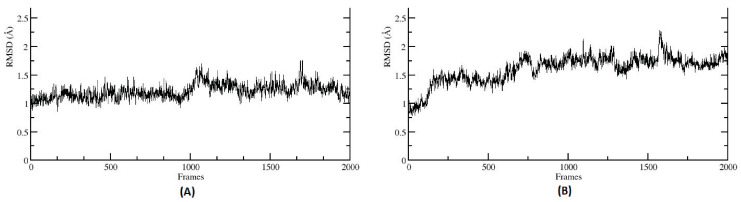
Root Mean Squared Deviation (RMSD) of selected metabolites’ complex with the CA family proteins. RMSD plot indicates a stable interaction for both the protein–ligand complexes (**A**) CA I and 3, 4-Dihydroxycinnamic acid (DHCA), (**B**) CA II and 3-hydroxyhippuric acid (HHPA).

In the current work, MD simulations were only performed on CA protein members with selected diet-derived blueberry metabolites, based on the strong binding energy (Table 3), predicted by AutoDock Vina [32]. All the simulations were conducted in a water box containing 150 mM ions to mimic a biological system. Furthermore, to capture the dynamic trends in metabolites binding to CAs in the presence of an explicit medium, the RMSD-based structural clustering of each metabolite/enzyme complex was performed, considering 10,000 frames taken at 10 ps intervals from the entire 100 ns MD trajectory individually. The Root Mean Square Deviation (RMSD) plot was generated for each of the CA-metabolite interaction to measure the stability of the complex throughout the simulation. The RMSD plot was calculated considering the backbone atoms of the protein and all atoms of the metabolite (ligand) over a MD trajectory time of 100 ns. Lower and stable RMSD values indicate that the complex is maintaining its structural integrity (Figure 7 and Supplementary Figure S5). Hydrogen bonds analysis was performed on these 10,000 frames, and a representative structure from a frame towards the end of simulation, where the RMSD is stable, has been selected for display in each case. The MD simulation of CA-I was conducted with the cluster-0 metabolite, 3,4 Dihydroxycinnamic acid (DHCA), the strongest binding metabolite obtained from AutoDock Vina. The results have shown that DHCA binds near to the Zn binding cavity of CA-I during the simulation with stable RMSD. The Zn binding cavity is surrounded by residues HIS64, HIS67, HIS94 and HIS200, which form a stable binding pocket for DHCA (Supplementary Figure S6A). Hydrogen bond interaction between DHCA and amino acid residues was observed within the cutoff range of 3.5 Å during the simulation run. A similar interaction pattern was reported by Supuru et. al., in an X-ray structural experimental study of sulfonamide inhibitors with CA I [49]. In addition to interacting with the HIS residues around the Zn, sulfonamide inhibitors formed an extended network of hydrogen bonds with residues THR199 and GLU106. In the same study, topiramate, a widely used antiepileptic drug, has been shown as an inhibitor of CA I with a binding site in the Zn binding pocket. A study of the crystal structure of topiramate with CA I has been shown to bind in the Zn binding pocket, forming a hydrogen bonds network with residues HIS64, HIS96, HIS110, HIS94, THR199 and GLU92 [49].

**Figure 8:**
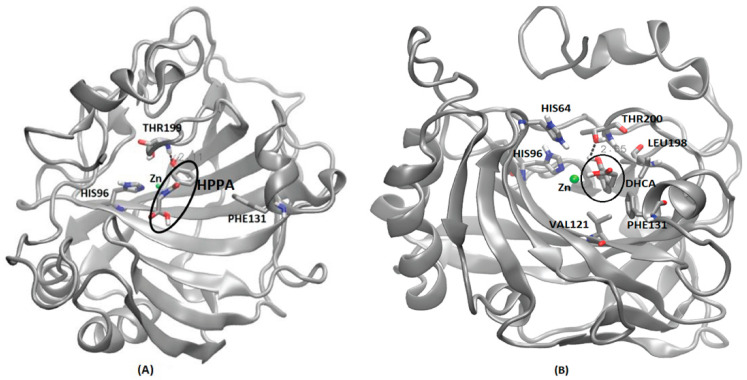
Interaction details of selected metabolites with CA family proteins after molecular dynamics simulations. (**A**) CA II and 3-hydroxyhippuric acid (HHPA), (**B**) CA VII and 3, 4-Dihydroxycinnamic acid (DHCA). Hydrogen bond is represented by dotted lines expressed in Å. HHPA and DHCA are highlighted in a dark circle.

Similar to CA I, DHCA also had the strongest binding affinity with CA VII based on the predicted AutoDock scores (Table 3). MD simulations have shown that DHCA has stable binding in the Zn binding cavity of CA-VII, which is reflected in the RMSD plot (Supplementary Figure S5E). DHCA shares the binding cavity near the Zn binding site, surrounded by the residues VAL121, HIS96, HIS64, THR200, LEU198 and PHE131 (Figure 8B). Unlike CA I, DHCA in the Zn binding cavity of CA VII forms a strong hydrogen bond (2.65 Å) with THR200 during the MD simulation run. Similar binding sites have been reported for various benzene sulphonamide inhibitors of CA VII in other computational and experimental studies, showing various interactions, along with hydrogen bonds, with THR199, HIS96, HIS119 and HIS94 [50,51].

Except CA I and CA VII, all other CAs showed the strongest binding affinity with 3-Hydroxyhippuric acid (HHPA) (Table 3). HHPA occupied a similar binding cavity in the CAs proteins, near the Zn binding cavity. CA II interaction with HHPA forms a strong hydrogen bond of 2.11 Å with THR199 and resides within the binding crevice between HIS64 and THR199 (Figure 8A). In a similar study [52], a brinzolamide molecule occupied the same binding pocket in the Zn residing cavity, surrounded by the residues HIS64, PHE131, PRO202 andTHR199. Unlike HHPA, brinzolamide was not reported to form any hydrogen bond-based interaction with the surrounding residues, except van der Waals contacts with PHE131 and PRO202. Similarly, in the case of CA III, HHPA resides in the Zn binding pocket, forming hydrogen bond interactions with ASN62 and THR200. The amino side chain of ASN62 forms a strong hydrogen bond (2.60 Å) with the carboxylic tail of 3-Hydroxyhippuric acid. Also, the THR200 side chain of CA III forms a strong hydrogen bond (1.99 Å) with 3-Hydroxyhippuric acid (Supplementary Figure S6B). A similar interaction for CA III’s interaction with histidylhistidine has been reported, where the imidazole ring of PHE198, along with residues LYS64, ASN67 and THR200, play an important role in ligand binding [53]. CA IV also displays a Zn binding cavity as a binding site for 3-Hydroxyhippuric acid, forming a hydrogen bond interaction with residues THR199 (2.21 Å), GLN92 (3.98 Å), ASN (2.22 Å), and THR200 (3.23 Å) during MD simulations (Supplementary Figure S6C). The experimental study of different CA IV inhibitors such as acetazolamide, benzenesulfomide, ethoxaolamide and topiramate, along with their derivatives, show that these compounds also occupy the Zn binding site, interacting with the residues TRP6, ASN69, HIS71, GLN96, HIS98, VAL125, ILE146, THR200, THR209 and TRP218 surrounding the Zn binding cavity [54]. As the human CA V was not available (either as an X-ray crystal or NMR structure), we have considered the murine CA V structure for this study. Our MD simulations show that 3-Hydroxyhippuric acid occupies the usual Zn binding cavity, with hydrogen bond interaction with THR199 (2.14 Å) (Supplementary Figure S6D). However, ligand binding studies of CA VA have not been reported so far in the literature. For CA VI, HHPA occupies the Zn binding site surrounded by the residues HIS94, HIS96, HIS119, ASN62, THR200 and THR199, forming a strong hydrogen bond (2.14 Å) interaction with THR199 (Supplementary Figure S6E). Direct evidence for the crystallographic structures of inhibitors binding to CA VI is not available; however, a site-directed mutagenesis of CA VI, called a CA VI-mimic, was shown to have bound a series of benzene sulfonamides in the Zn binding pocket, as is typical for all the other CAs [55]. In the case of CA IX, MD simulations shows that HHPA was not able to bind in the binding pocket near Zn binding site as seen in other CAs. The ligand is embedded between the loops in the protein surrounded by the residues GLY365, PRO366, GLN372, ASN374, GLY144 and LEU 371 (Supplementary Figure S6F). MD simulations have shown that the highly unstructured c-terminal region, along with the loops surrounding the Zn embedded region, might have prevented the ligand from entering the Zn binding site. However, in an experimental study, it was shown that various sulfonamides and their derivatives interact with CA IX through a binding mechanism that involves a binding site coordinated by the Zn ion in the protein’s active site. Crucial amino acid residues in CA IX for sulfonamides’ ligand binding include HIS94, HIS96, and HIS119, which coordinate the Zn ion, and THR200, which interacts with the sulfonamide group of the inhibitor, involves both the protein and the ligand undergoing protonation–deprotonation reactions [56]. To verify our MD simulations, further studies that involve Steered Molecular Dynamics will be applied to understand the entry and exit mechanisms of metabolites in CA IX, similar to our previous studies [33,34,35,37]. For CA XII, HHPA occupies the Zn binding site of protein surrounded by the residues THR199, THR200, SER132 and LEU198, with which the side chain of THR199 forms a hydrogen bond with 3-Hydroxyhippuric acid (Supplementary Figure S6G). Similar interactions were reported in experimental studies, where ligands, such as sulfonamides and their derivatives, interact with CA XII through a binding mechanism that involves a binding site coordinated by the Zn ion in the protein’s active site. Key amino acid residues in CA XII crucial for ligand binding include His94, His96, and His119, which coordinate the Zn, similar to CA IX, but with additional involvement from residues such as Thr198, which interacts with the sulfonamide group of the inhibitor [56]. For CA XIII, MD simulations show that HHPA binds in the Zn binding pocket surrounded by the residues HIS94, HIS96, HIS119 and THR199 (Supplementary Figure S6H), similar to the experimental results, where acetazolamide was reported to bind CA-XII primarily through interactions involving the Zn ion at the active site. The key residues crucial for this binding are HIS94, HIS96, and HIS119, which coordinate the Zn ion, and THR200, which is important for stabilizing the inhibitor’s binding [57]. In the case of CA XIV, HHPA binds near the Zn binding site surrounded by the residues HIS119, HIS94, HIS96 and THR199 (Supplementary Figure S6I). Our MD simulation results are similar to the experimental study, where it has been established that acetazolamide, a well-known inhibitor of CA enzymes, interacts with CA-XIV primarily through several key residues. The crystallographic studies reveal that acetazolamide binds in the active site of CA XIV form crucial interactions with residues such as THR199, GLU106, and HIS64 [52]. These residues are pivotal in stabilizing the sulfonamide group of acetazolamides through hydrogen bonds and van der Waals interactions. Compared to other CAs like CA II and CA IX, the binding affinity of acetazolamide to CA XIV shows slight variations due to differences in the spatial arrangement and chemical environment of these active site residues. For instance, while the interaction with THR199 is conserved across many CA isozymes, the specific role of GLU106 and HIS64 can vary, leading to differences in binding strength and inhibitor efficiency [52]. Although CA proteins are present in both cytosolic and membrane-bound forms, the PDB structures used in our study primarily include only the soluble extracellular domains of these proteins, excluding the membrane-associated parts. Within the membrane-associated CAs, CA IV and CA XV are glycosylphosphatidylinositol (GPI)-anchored, while CA IX, CA XII, and CA XIV are transmembrane proteins [58]. In future studies, it will be important to consider the membrane-associated nature of these proteins when modeling their actual mechanisms, as this can impact their functional interactions and structural features. Our study was limited to predicting the binding site and its stability using MD simulations, without incorporating the membrane-associated components.

Overall, the MD simulations of CA proteins with diet-derived blueberry metabolites, specifically DHCA and HHPA, demonstrated stable interactions at the Zn-binding cavities of several CA isozymes. RMSD plots indicated that these complexes maintained structural integrity throughout the 100 ns simulation, except for CA VII-DHCA, where there was a steady increase in the RMSD towards the end of simulation (Supplementary Figure S5E). However, DHCA showed strong binding affinity and stable interactions near the Zn-binding site of CA I and CA VII, forming crucial hydrogen bonds with residues like HIS64, HIS96, and THR200. Similarly, HHPA exhibited stable binding in the Zn-binding pockets of CA II, CA III, CA IV, and CA VI, forming significant hydrogen bonds with residues such as THR199, ASN62, and GLN92. However, HHPA failed to bind near the Zn site in CA IX, likely due to the highly unstructured c-terminal region and surrounding loops. The consistent binding patterns and interaction residues identified in these simulations align well with experimental studies, highlighting the potential of these metabolites in modulating CA activity and providing a basis for future targeted studies. Additionally, the metabolite DHCA showed the highest bioactivity scores, whereas HHPA has shown high to moderate bioactivity scores among the tested compounds, indicating their strong potential as bioactive agents. These scores suggest that DHCA and HHPA are promising candidates for further investigation, due to their significant binding affinities and potential biological effects. Additionally, the pathway analysis identified that these metabolites are involved in critical metabolic and signaling pathways related to CA function, and molecular dynamics simulations demonstrated that both DHCA and HHPA maintain stable interactions with CA isozymes, particularly in the Zn-binding cavities. This stability is crucial for understanding how these metabolites might modulate enzyme activity in vivo, supporting their potential therapeutic roles. Although experimental investigations directly probing the interaction between CAs and blueberry metabolites are currently limited, emerging evidence suggests the potential inhibitory effects of blueberry extract on CA, thereby holding pharmaceutical relevance [26]. Computational analyses have recently implicated caffeic acid in interactions with CA, revealing a significant reduction in energy expenditure during these engagements, hinting at a therapeutic role for this phenolic compound [59]. In a separate study, the inhibitory effects of CAPE (caffeic acid phenyl ester) on various human CA isoforms (I, II, IX, and XII) were explored, with CAPE demonstrating inhibitory effects and dissociation constants (K_d_) [60]. Beyond the CA protein family, our prior research has established the interaction of hippuric acid with HCAR1 and HCAR2, displaying a higher affinity for HCAR1 [11,14]. Moreover, other studies have reported the association of esters of 4-hydroxybenzoic acid with estrogen receptors, albeit with low binding affinity [61].

As CA proteins are enzymes that play a crucial role in regulating pH and fluid balance in various tissues, metabolites from a blueberry-rich diet may have potential in inhibiting these proteins, leading to significant biological effects. For instance, certain CA isoforms, such as CA IX and CA XII, are overexpressed in various cancers [62]. These isoforms help tumor cells survive in acidic microenvironments by regulating pH conditions. Inhibiting these CAs can disrupt the pH balance, leading to a less favorable environment for cancer cell survival and growth. Blueberry metabolites, which have antioxidant and anti-inflammatory properties, may enhance these effects, potentially contributing to cancer prevention or therapy. Additionally, the metabolites found in blueberries, such as quercetin, myricetin, and resveratrol, have multiple biological activities [63,64]. When combined with CA inhibition, these compounds could have synergistic effects that can work alongside CA inhibition to reduce oxidative stress and protect tissues from damage, thereby enhancing the overall impact on health. CA inhibitors have also been studied for their neuroprotective properties, particularly in conditions like glaucoma and epilepsy [65]. Blueberries contain compounds like anthocyanins and flavonoids, which have been shown to cross the blood–brain barrier and exhibit neuroprotective effects [66]. By inhibiting CA activity, these metabolites could help in reducing intraocular pressure in glaucoma or modulating neuronal excitability in epilepsy [67]. Additionally, CA inhibitors can impact blood pressure regulation and vascular function, contributing to cardiovascular health. CAs play a role in metabolic processes and respiratory function [68]. Inhibiting CAs in certain tissues can help regulate metabolic pathways and improve respiratory efficiency. Blueberry metabolites might enhance these effects, supporting metabolic health and potentially benefiting conditions like metabolic syndrome or chronic obstructive pulmonary disease (COPD) [69,70,71].

Our study uses computational methods to predict protein targets for metabolites, which introduces multiple layers of uncertainty, such as the accuracy of the computational models, the quality of input data, and the interpretation of predicted interactions, especially crucial when dealing with large datasets. Additionally, elevated compound levels observed in metabolomic profiles can result from several factors beyond direct metabolic derivation, including the native presence of compounds in the diet (e.g., blueberries) or their formation through alternative metabolic pathways influenced by dietary components or their metabolites. This distinction highlights the complexity of interpreting metabolomic data and emphasizes that while our findings suggest possible mechanisms for the physiological effects of blueberry-derived compounds, they should be viewed as preliminary. These predictions provide a foundation for further experimental validation and hypothesis generation but must be interpreted with caution in broader contexts where different factors may influence metabolite levels and interactions. Acknowledging these limitations ensures a balanced perspective on the conclusions and encourages further research to substantiate the computational predictions with empirical data. To validate our predictions and enhance their reliability, future work will involve the integration of additional computational tools, such as SwissADME [72] and biotransformation prediction models [73]. SwissADME assess the pharmacokinetics, drug-likeness, and medicinal chemistry friendliness of the predicted compounds, providing insights into their potential efficacy and bioavailability. Biotransformation predictions will further elucidate how these metabolites are processed in the body, offering a more comprehensive understanding of their metabolic pathways and interactions. By combining these advanced computational approaches, we aim to refine our predictions and reduce the risk of false positives, paving the way for more accurate and experimentally validated insights into the biological effects of blueberry-derived metabolites.

Future studies will involve selecting prospective metabolites and proteins for a more in-depth exploration. Further, metabolites exhibiting promising behavior in MD simulations will be prioritized for subsequent experimental investigations, employing techniques such as Circular Dichroism (CD) and Isothermal Titration Calorimetry (ITC). These experimental studies will encompass both in vitro and in vivo assessments, contributing to a more comprehensive understanding of the identified metabolites and their interactions with proteins. The potential of this approach can be further harnessed by extending its application to delve into a more expansive array of metabolites or bio signatures and diverse biological contexts. Expanding the scope to encompass a broader spectrum of metabolites following untargeted metabolomics and will offer a comprehensive perspective on their interactions with proteins, enabling a nuanced understanding of cellular processes. Additionally, the exploration of tissue-specific responses presents an exciting avenue for future research, as different tissues may exhibit distinct metabolic profiles and responses to dietary interventions. The horizon of inquiry extends to investigating a myriad of dietary interventions beyond blueberry supplemented diets, as explored in this present study. Analyzing the effects of various dietary components on metabolite–protein interactions could unveil specific molecular mechanisms and pathways influenced by different nutritional stimuli. This, in turn, could provide valuable insights into tailoring dietary strategies for health optimization and disease prevention, propelling science one step further in realizing precision nutrition and health. By elucidating the intricacies of metabolite–protein interactions, this approach lays the foundation for innovative strategies to address complex biological challenges. These strategies may extend beyond dietary considerations, encompassing the development of targeted therapeutics and precision medicine approaches that leverage the knowledge gained from unraveling the dynamic interplay between metabolites and proteins in diverse biological contexts.

## 4. Conclusions

In this investigation, we utilized structure-based target prediction for a selected group of dietary relevant metabolites. Particularly, the metabolites derived from a blueberry diet were found to interact significantly with CA protein families, suggesting their involvement in various physiological processes, such as acid-base regulation, respiration, fluid balance, bone metabolism, neurotransmission, and specific cellular metabolic pathways. This finding implies that these diet-derived metabolites may have a wide range of biological effects, mediated through CA inhibition or modulation. The broad-spectrum inhibitors of CAs, like those identified in our study, may therefore influence multiple biological pathways. Beyond CAs, our predictions highlighted other noteworthy protein targets, including D(1A) dopamine receptor, epidermal growth factor receptor, estrogen receptor beta, and macrophage migration inhibitory factor. This comprehensive workflow, as demonstrated in this study, holds potential applicability to any dataset of small molecules. While the Cluster-1 metabolites did not exhibit high-confidence predictions for specific protein targets, it is conceivable that these metabolites may exert influence at the gene level, potentially affecting RNA and DNA processes. The subsequent phases of our research will encompass an exploration of gene-level interactions with these metabolites, aiming to identify and elucidate their impact on genetic mechanisms. The computational workflow employed in screening potential targets using this limited set of metabolites has yielded promising outcomes, shedding light on crucial metabolic pathways, and allowed for piloting a workflow which can be applied to untargeted metabolomics’ “dark matter” discovery in future precision nutrition and health research pursuits.

## Figures and Tables

**Table 1:** List of diet-derived metabolites (Cluster-0 and Cluster-1) in human subjects following a blueberry diet intervention, predicted bioactivity scores and their protein targets. Out of the 27 metabolites, 15 were grouped into Cluster-0, and the remaining 12 metabolites were grouped into Cluster-1 metabolites. Molinspiration bioactivity scores for Cluster-0 and Cluster-1 metabolites for GPCR ligand, channel, kinase inhibitor, nuclear receptor ligand, protease inhibitor and enzyme inhibitor activity. Metabolites with scores greater than 0 are deemed highly active, those with scores between 0 and −0.5 are considered moderately active, and metabolites with scores less than −0.5 are classified as inactive for the respective categories. Highly active compounds are highlighted in green and moderately active compounds are highlighted in yellow. The predominant trend observed within Cluster-0 metabolites is their interaction with carbonic anhydrase family proteins, indicated by 90–100% confidence hits, in addition to interactions with various other proteins. (CA—carbonic anhydrase; D1A—dopamine receptor; EGFR—epidermal growth factor receptor; AR—aldose reductase; ERβ—estrogen receptor beta; HCAR1—human carboxylic acid receptor 1, HCAR2—human carboxylic acid receptor 2).

Compounds (Cluster-0)	GPCR Ligand Activity	Channel Activity	Kinase Inhibitor Activity	Protease Inhibitor Activity	Enzyme Inhibitor Activity	Nuclear Receptor Ligand Activity	Protein Target (90–100% Confidence Hit)
4-Hydroxybenzoic acid	−0.98	−0.93	−1.21	−1.19	−0.41	−0.62	CA I, CA II, CA III, CA IV, (D1A), EGFR
3,4-Dihydroxyhydrocinnamic acid	−0.29	−0.06	−0.79	−0.52	0.01	−0.12	-
3-(4-Hydroxyphenyl) propionic acid (Desaminotyrosine)	−0.35	−0.07	−0.89	−0.57	−0.04	−0.14	-
3,4-Dihydroxybenzeneacetic acid	−0.46	−0.06	−0.92	−0.64	−0.04	−0.16	-
Hippuric acid	−0.52	−0.2	−0.87	−0.39	−0.19	−0.76	CA II, CA III, CA IX, HCAR2
3,5-Dihydroxybenzoic acid	−0.86	−0.34	−1.04	−1.08	−0.31	−0.47	CA I, CA II, CA VII, CA XII, CA XIV, CA IX
3,4-dihydroxybenzoic acid (Protocatechuic acid)	−0.88	−0.35	−1.1	−1.09	−0.34	−0.58	CA I, CA II, CA VII, CA VI, CA XII, CA XIV, CA IX, CA IV
2,4-Dihydroxybenzoic acid	−0.81	−0.33	−0.99	−1.02	−0.28	−0.5	CA I, CA II, CA XII
4-Hydroxycinnamic acid	−0.56	−0.26	−0.91	−0.87	−0.15	−0.12	AR, CA I, CA II, CA III, CA VI, CA VII, CA XII, ERβ
2,3-Dihydroxybenzoic acid (2-Pyrocatechuic acid)	−0.8	−0.24	−1.02	−1.06	−0.3	−0.74	-
2,4,6-Trihydroxybenzaldehyde	−0.96	−0.3	−0.82	−1.33	−0.46	−0.59	-
2-Hydroxybenzoic acid (Salicylic acid)	−0.98	−0.43	−1.22	−1.14	−0.41	−0.79	CA I, CA II, CA XII
3-Hydroxybenzoic acid	−0.99	−0.42	−1.25	−1.21	−0.43	−0.61	CA I, CA II, CA VI, CA XII, CA IX, HCAR1, HCAR2
3-Hydroxyhippuric acid	−0.37	−0.12	−0.7	−0.33	−0.07	−0.42	-
4-Hydroxybenzyl alcohol	−1.91	−1.2	−2.02	−2.02	−1.31	−1.78	-
**Compounds** **(Cluster-1)**							
4-Hydroxy-3-methoxyphenylacetic acid (Homovanillic acid)	−0.65	−0.28	−0.69	−0.82	−0.15	−0.44	-
4-Hydroxy-3,5-dimethoxybenzoic acid (Syringic acid)	−0.29	−0.14	0	−0.27	−0.62	−0.74	CA I, CA II, CA III, CA VII
Chlorogenic acid (3-Caffeoylquinic acid)	−0.41	−0.24	−0.93	−0.59	−0.16	−0.29	-
3-Methoxybenzenepropanoic acid	−0.29	−0.14	−0.7	−0.56	−0.03	−0.16	-
3-(3-hydroxy-4-methoxyphenyl) propanoic acid	−0.47	−0.3	−0.72	−0.81	−0.12	−0.14	-
4-Hydroxy-3-methoxycinnamic acid (Ferulic acid)	−0.71	−0.32	−0.82	−0.95	−0.25	−0.56	CA II, CA VII
3,5-Dihydroxy-4-methoxybenzoic acid (4-O-Methylgallic acid)	−0.29	−0.14	−0.7	−0.56	−0.03	−0.16	-
3-(4-Hydroxy-3-methoxyphenyl) propanoic acid (Hydroferulic acid)	−0.85	−0.42	−0.99	−1.12	−0.35	−0.61	-
3-Hydroxy-4-methoxybenzoic acid	−0.32	−0.2	−0.47	−0.56	0.03	−0.03	-
4-Hydroxy-3,5-dimethoxycinnamic acid (Sinapic acid)	−0.85	−0.42	−0.99	−1.12	−0.35	−0.61	-
4-Hydroxy-3-methoxybenzoic acid (Vanillic acid)	−1.13	−0.51	−1.22	−1.31	−0.62	−0.88	-
4-Hydroxybenzoic acid methyl ester (Methylparaben)	−0.23	−0.01	−0.78	−0.53	0.1	−0.08	CA VII, CAXII

**Table 2:** Predicted binding energies for experimentally known compounds interacting with carbonic anhydrase family proteins. The Autodock Vina docking energies for known compounds that bind with carbonic anhydrase family proteins, as adapted from [46], are expressed in kcal/mol.

Compounds	CA-I	CA-II	CA-III	CA-IV	CA-VA	CA-VI	CA-VII	CA-IX	CA-XII	CA-XIII	CA-XIV
Adrenaline	−6.2	−5.8	−5.7	−5.5	−6.1	−6.1	−6.1	−6.4	−6	−5.7	−5.9
Dopa	−6.6	−6.3	−6.5	−5.5	−6.6	−6.5	−6.5	−6.9	−6.3	−5.9	−6.2
Dopamine	−6.2	−5.6	−5.5	−5.2	−6.1	−6	−6.2	−6.2	−5.8	−5.5	−6
Histamine	−4.5	−4.5	−3.9	−4.2	−4.1	−4.7	−4.3	−4.8	−4.4	−4.3	−4.3
Histidine	−5.3	−5.1	−5	−5	−5.3	−5.3	−5.4	−5.8	−5.2	−5.3	−5.3
Phenylalanine	−5.8	−6.1	−6.3	−5.6	−5.7	−6	−5.8	−6.3	−6.2	−5.5	−6.4
Serotonine	−6.1	−6.1	−5.5	−5.4	−6	−6.4	−6.1	−7	−6.2	−5.8	−6.1
Tryptophan	−6.4	−6.6	−6.4	−6.1	−6.6	−7	−6.6	−7.2	−6.8	−6.3	−6.7
Tyrosine	−6.1	−6	−6.2	−5.5	−6	−6	−5.8	−6.7	−6	−5.7	−6.2
Acetazolamide	−6.1	−6.2	−5.6	−5.6	−6.3	−6.7	−6.1	−6.3	−5.8	−5.9	−6.2
Brinzolamide	−6.2	−7.1	−6.0	−6.2	−6.7	−6.9	−6.6	−6.4	−6.3	−6.5	−6.9
Celecoxib	−7.8	−8.2	−7.8	−7.8	−8.8	−8.6	−7.5	−8.3	−8.9	−8.0	−8.4
COUMATE	−7.0	−7.6	−7.0	−6.7	−7.2	−7.5	−7.4	−7.4	−7.2	−6.5	−7.5
Dichlorophenamide	−6.3	−6.6	−6.2	−6.6	−7.1	−6.7	−6.2	−7.0	−6.8	−5.8	−7.0
Dorzolamide	−6.5	−6.3	−6.8	−6.5	−7.6	−7.6	−6.7	−6.8	−7.0	−6.6	−7.7
EMATE	−8.5	−8.3	−7.5	−7.6	−8.1	−8.4	−8.1	−8.5	−7.4	−7.5	−8.1
Ethoxzolamide	−6.1	−6.1	−5.9	−5.8	−6.4	−6.5	−6.6	−6.8	−6.1	−5.9	−6.4
Indisulam	−7.9	−8.5	−7.4	−7.6	−8.3	−9.2	−8.1	−8.9	−7.8	7.5	−8.7
Methazolamide	−5.6	−6.4	−5.6	−5.7	−6.4	−6.4	−6.2	−6.4	−6.6	−5.6	−6.3
Saccharin	−6.4	−6.3	−5.9	−6.3	−6.9	−6.8	−6.8	−7.0	−6.2	−5.8	−7.1
Sulpiride	−7.0	−7.1	−6.7	−6.7	−7.4	−7.4	−6.5	−7.5	−7.3	−6.5	−7.4
Sulthiame	−7.5	−7.1	−6.6	−6.4	−6.8	−7.6	−7.2	−7.3	−7.2	−6.1	−7.3
Topiramate	−7.1	−7.2	−7.2	−6.7	−6.8	−7.0	−6.3	−7.9	−7.6	−6.6	−7.5
Valdecoxib	−7.3	−8.2	−7.7	−7.7	−7.7	−8.7	−7.4	−8.2	−8.3	−8.0	−8.2
Zonisamide	−6.9	−6.9	−6.8	−6.4	−6.6	−7.1	−7.0	−6.9	−6.6	−6.0	−7.0

**Table 3:** Docking energy of Cluster-0 metabolites with carbonic anhydrases (CA) family proteins. Predicted binding energy values of Cluster-0 metabolites with various carbonic anhydrase family proteins. Binding energy is expressed in kcal/mol. The protein-metabolite interactions were selected from the highest binding energies listed across the table highlighted in bold and underline.

Cluster-0 Metabolites	CA I	CA II	CA III	CA IV	CA VA	CA VI	CA VII	CA IX	CA XII	CA XIII	CA XIV
2,4,6-trihydroxybenzaldehyde	−5.6	−5.7	−5.2	−5.2	−5.4	−5.5	−5.6	−6.7	−5.2	−4.9	−5.9
2,4-Dihydroxybenzoic acid	−6.1	−6.2	−5.4	−5.4	−5.8	−6.2	−6.3	−6.8	−5.7	−5.4	−5.9
2,3-Dihydroxybenzoic acid (2-Pyrocatechuic acid)	−6.3	−6.1	−6.0	−5.5	−6.0	−6.4	−6.5	−6.8	−5.8	−5.6	−6.0
3,4-Dihydroxybenzeneacetic acid	−6.4	−5.9	−5.8	−5.4	−6.3	−6.1	−6.5	−6.6	−6.4	−5.7	−6.2
3,4-Dihydroxyhydrocinnamic acid	**−6.6**	−6.1	−6.2	−5.6	−6.3	−6.3	**−6.6**	−7.0	−6.3	−5.9	−6.4
3,5-Dihydroxybenzoic acid	−6.1	−5.8	−5.4	−5.5	−5.8	−6.1	−5.7	−6.7	−5.6	−5.2	−5.6
3-Hydroxybenzoic acid	−6.0	−5.7	−5.8	−5.6	−5.8	−6.1	−5.9	−6.4	−5.7	−5.5	−5.8
3-Hydroxyhippuric acid	−6.3	**−6.9**	**−6.8**	**−6.3**	**−6.8**	**−6.7**	−6.3	**−7.5**	**−6.4**	**−6.4**	**−6.5**
4-Hydroxybenzoic acid	−5.8	−5.8	−5.3	−5.2	−5.6	−5.8	−5.4	−6.2	−5.2	−5.3	−5.6
4-Hydroxybenzyl alcohol	−5.4	−5.3	−5.2	−4.8	−5.3	−5.2	−5.1	−5.9	−4.9	−5.0	−5.0
4-Hydroxycinnamic acid	−5.9	−5.7	−6.1	−5.8	−5.7	−5.8	−5.7	−7.2	−5.9	−5.9	−6.0
3-(4-Hydroxyphenyl) propionic acid (Desaminotyrosine)	−6.1	−5.7	−5.9	−5.4	−5.7	−6.1	−5.7	−6.9	−5.8	−5.7	−6.0
2-Benzamidoacetic acid (Hippuric acid)	−5.9	−6.6	−6.7	−6.1	−6.6	−6.6	−6.0	−6.7	−6.2	−6.2	−6.5
3,4-Dihydroxybenzoic acid (Protocatechuic acid)	−6.4	−6.1	−5.6	−5.4	−6.3	−6.5	−6.4	−6.4	−6.1	−5.7	−6.1
2-Hydroxybenzoic acid (Salicylic acid)	−5.9	−6.0	−5.8	−5.5	−5.9	−6.2	−6.3	−6.3	−5.6	−5.4	−5.9

## Data Availability

Data is not available in public domain. However, the entire raw data and processed data is stored in our local high performance computing cluster. Data is available upon request.

## Supplementary Materials

The following supporting information can be downloaded at: https://www.mdpi.com/article/10.3390/metabo14080430/s1, Supplementary Figure S1: Two-dimensional structure of Cluster-0 metabolites along with their names obtained from PubChem database. Supplementary Figure S2: Two-dimensional structure of Cluster-1 metabolites along with their names obtained from PubChem database. Supplementary Figure S3: Docked state of metabolites. (A) 3-hydroxyhippuric acid with estrogen receptor beta, (B) 3,4-dihydroxyhippuric acid with epidermal growth factor receptor, (C) 3-hydroxyhippuric acid with macrophage migration inhibitory factor, (D) Hippuric acid with dopamine receptor. Docked metabolites are highlighted in the red-colored circle. Supplementary Figure S4: Two-dimensional structure of known compounds, along with their names obtained from PubChem database, that bind with CA family proteins. Compounds were adapted from Supuran et al. [46]. Supplementary Figure S5: Root Mean Squared Deviation (RMSD) of selected metabolites, 3-hydroxyhippuric acid (HHPA) and 3, 4-Dihydroxycinnamic acid (DHCA) complex with all the CA family proteins. (A) CA III-HHPA, (B) CA IV-HHPA, (C) CA VA-HHPA, (D) CA VI-HHPA, (E) CA VII-DHCA, (F) CA IX-HHPA, (G) CA XII-HHPA, (H) CA XIII-HHPA, (I) CA XIV-HHPA. Supplementary Figure S6: Interaction details of selected metabolites, 3-hydroxyhippuric acid (HHPA) and 3, 4-Dihydroxycinnamic acid (DHCA) with CA family proteins after molecular dynamics simulations. (A) CA I-DHCA, (B) CA III-HHPA, (C) CA IV-HHPA, (D) CA VA-HHPA, (E) CA VI-HHPA, (F) CA IX-HHPA, (G) CA XII-HHPA, (H) CA XIII-HHPA, (I) CA XIV-HH.PA. Supplementary Table S1. Protein targets for Cluster-0 metabolites predicted from SWISS target prediction. Supplementary Table S2. Protein targets for Cluster-0 metabolites predicted from SWISS target prediction. Supplementary Table S3. Docking scores of Cluster-0 metabolites with four different proteins.

## References

[1] Judge A., Dodd M.S. (2020). Metabolism. Essays Biochem..

[2] Clish C.B. (2015). Metabolomics: An Emerging but Powerful Tool for Precision Medicine. Mol. Case Stud..

[3] Tan S.Z., Begley P., Mullard G., Hollywood K.A., Bishop P.N. (2016). Introduction to Metabolomics and Its Applications in Ophthalmology. Eye.

[4] Ellis D.I., Dunn W.B., Griffin J.L., Allwood J.W., Goodacre R. (2007). Metabolic Fingerprinting as a Diagnostic Tool. Pharmacogenomics.

[5] Roberts L.D., Souza A.L., Gerszten R.E., Clish C.B. (2012). Targeted Metabolomics. Curr. Protoc. Mol. Biol..

[6] Dudley E., Yousef M., Wang Y., Griffiths W.J. (2010). Targeted Metabolomics and Mass Spectrometry. Adv. Protein Chem. Struct. Biol..

[7] Qiu S., Cai Y., Yao H., Lin C., Xie Y., Tang S., Zhang A. (2023). Small Molecule Metabolites: Discovery of Biomarkers and Therapeutic Targets. Signal Transduct. Target. Ther..

[8] Qiu S., Cai Y., Wang Z., Xie Y., Zhang A. (2023). Decoding Functional Significance of Small Molecule Metabolites. Biomed. Pharmacother..

[9] DeBerardinis R.J., Keshari K.R. (2022). Metabolic Analysis as a Driver for Discovery, Diagnosis, and Therapy. Cell.

[10] Sliwoski G., Kothiwale S., Meiler J., Lowe E.W. (2014). Computational Methods in Drug Discovery. Pharmacol. Rev..

[11] Chen J.-R., Zhao H., Wankhade U.D., Chintapalli S.V., Li C., Gai D., Shankar K., Zhan F., Lazarenko O.P. (2021). GPR109A Mediates the Effects of Hippuric Acid on Regulating Osteoclastogenesis and Bone Resorption in Mice. Commun. Biol..

[12] Chen J.-R., Lazarenko O.P., Zhang J., Blackburn M.L., Ronis M.J.J., Badger T.M. (2014). Diet-Derived Phenolic Acids Regulate Osteoblast and Adipocyte Lineage Commitment and Differentiation in Young Mice. J. Bone Miner. Res..

[13] Chen J.-R., Lazarenko O.P., Wu X., Kang J., Blackburn M.L., Shankar K., Badger T.M., Ronis M.J.J. (2010). Dietary-Induced Serum Phenolic Acids Promote Bone Growth via P38 MAPK/β-Catenin Canonical Wnt Signaling. J. Bone Miner. Res..

[14] Bhandari D., Kachhap S., Madhukar G., Adepu K.K., Anishkin A., Chen J.R., Chintapalli S.V. (2022). Exploring GPR109A Receptor Interaction with Hippuric Acid Using MD Simulations and CD Spectroscopy. Int. J. Mol. Sci..

[15] Curtis P.J., Berends L., van der Velpen V., Jennings A., Haag L., Chandra P., Kay C.D., Rimm E.B., Cassidy A. (2022). Blueberry Anthocyanin Intake Attenuates the Postprandial Cardiometabolic Effect of an Energy-Dense Food Challenge: Results from a Double Blind, Randomized Controlled Trial in Metabolic Syndrome Participants. Clin. Nutr..

[16] Curtis P.J., van der Velpen V., Berends L., Jennings A., Feelisch M., Umpleby A.M., Evans M., Fernandez B.O., Meiss M.S., Minnion M. (2019). Blueberries Improve Biomarkers of Cardiometabolic Function in Participants with Metabolic Syndrome—Results from a 6-Month, Double-Blind, Randomized Controlled Trial. Am. J. Clin. Nutr..

[17] Kim S., Thiessen P.A., Bolton E.E., Chen J., Fu G., Gindulyte A., Han L., He J., He S., Shoemaker B.A. (2016). PubChem Substance and Compound Databases. Nucleic Acids Res..

[18] Gaulton A., Bellis L.J., Bento A.P., Chambers J., Davies M., Hersey A., Light Y., McGlinchey S., Michalovich D., Al-Lazikani B. (2012). ChEMBL: A Large-Scale Bioactivity Database for Drug Discovery. Nucleic Acids Res..

[19] Chen J., Swamidass S.J., Dou Y., Bruand J., Baldi P. (2005). ChemDB: A Public Database of Small Molecules and Related Chemoinformatics Resources. Bioinformatics.

[20] Wishart D.S., Guo A., Oler E., Wang F., Anjum A., Peters H., Dizon R., Sayeeda Z., Tian S., Lee B.L. (2022). HMDB 5.0: The Human Metabolome Database for 2022. Nucleic Acids Res..

[21] Leite T.B., Gomes D., Miteva M.A., Chomilier J., Villoutreix B.O., Tuffery P. (2007). Frog: A FRee Online DruG 3D Conformation Generator. Nucleic Acids Res..

[22] Case D.A., Cheatham T.E., Darden T., Gohlke H., Luo R., Merz K.M., Onufriev A., Simmerling C., Wang B., Woods R.J. (2005). The Amber Biomolecular Simulation Programs. J. Comput. Chem..

[23] Van Der Spoel D., Lindahl E., Hess B., Groenhof G., Mark A.E., Berendsen H.J.C. (2005). GROMACS: Fast, Flexible, and Free. J. Comput. Chem..

[24] Land H., Humble M.S., Bornscheuer U., Höhne M. (2018). YASARA: A Tool to Obtain Structural Guidance in Biocatalytic Investigations. Protein Engineering. Methods in Molecular Biology.

[25] Prada Gori D.N., Llanos M.A., Bellera C.L., Talevi A., Alberca L.N. (2022). IRaPCA and SOMoC: Development and Validation of Web Applications for New Approaches for the Clustering of Small Molecules. J. Chem. Inf. Model..

[26] Li Y., Hu Z., Huo R., Cui Z. (2023). Preparation of an Indicator Film Based on Pectin, Sodium Alginate, and Xanthan Gum Containing Blueberry Anthocyanin Extract and Its Application in Blueberry Freshness Monitoring. Heliyon.

[27] Nieman D.C., Gillitt N.D., Chen G.-Y., Zhang Q., Sha W., Kay C.D., Chandra P., Kay K.L., Lila M.A. (2020). Blueberry and/or Banana Consumption Mitigate Arachidonic, Cytochrome P450 Oxylipin Generation During Recovery from 75-Km Cycling: A Randomized Trial. Front. Nutr..

[28] Liu T., Lin Y., Wen X., Jorissen R.N., Gilson M.K. (2007). BindingDB: A Web-Accessible Database of Experimentally Determined Protein-Ligand Binding Affinities. Nucleic Acids Res..

[29] Daina A., Michielin O., Zoete V. (2019). SwissTargetPrediction: Updated Data and New Features for Efficient Prediction of Protein Targets of Small Molecules. Nucleic Acids Res..

[30] Kanehisa M. (2000). KEGG: Kyoto Encyclopedia of Genes and Genomes. Nucleic Acids Res..

[31] Kuhn M., von Mering C., Campillos M., Jensen L.J., Bork P. (2007). STITCH: Interaction Networks of Chemicals and Proteins. Nucleic Acids Res..

[32] Trott O., Olson A.J. (2010). AutoDock Vina: Improving the Speed and Accuracy of Docking with a New Scoring Function, Efficient Optimization, and Multithreading. J. Comput. Chem..

[33] Adepu K.K., Kachhap S., Anishkin A., Chintapalli S.V. (2021). Structural and Energetic Insights into the Interaction of Niacin with the GPR109A Receptor. Bioinform. Biol. Insights.

[34] Adepu K.K., Bhandari D., Anishkin A., Adams S.H., Chintapalli S.V. (2022). Myoglobin–Pyruvate Interactions: Binding Thermodynamics, Structure–Function Relationships, and Impact on Oxygen Release Kinetics. Int. J. Mol. Sci..

[35] Adepu K.K., Bhandari D., Anishkin A., Adams S.H., Chintapalli S.V. (2022). Myoglobin Interaction with Lactate Rapidly Releases Oxygen: Studies on Binding Thermodynamics, Spectroscopy, and Oxygen Kinetics. Int. J. Mol. Sci..

[36] Watts F.M., Pouland T., Bunce R.A., Berlin K.D., Benbrook D.M., Mashayekhi M., Bhandari D., Zhou D. (2018). Activity of Oxygen-versus Sulfur-Containing Analogs of the Flex-Het Anticancer Agent SHetA2. Eur. J. Med. Chem..

[37] Chintapalli S.V., Jayanthi S., Mallipeddi P.L., Gundampati R., Suresh Kumar T.K., Van Rossum D.B., Anishkin A., Adams S.H. (2016). Novel Molecular Interactions of Acylcarnitines and Fatty Acids with Myoglobin. J. Biol. Chem..

[38] Humphrey W., Dalke A., Schulten K. (1996). VMD: Visual Molecular Dynamics. J. Mol. Graph..

[39] MacKerell A.D., Bashford D., Bellott M., Dunbrack R.L., Evanseck J.D., Field M.J., Fischer S., Gao J., Guo H., Ha S. (1998). All-Atom Empirical Potential for Molecular Modeling and Dynamics Studies of Proteins. J. Phys. Chem. B.

[40] Rousseeuw P.J. (1987). Silhouettes: A Graphical Aid to the Interpretation and Validation of Cluster Analysis. J. Comput. Appl. Math..

[41] Hernández-Hernández S., Ballester P.J. (2023). On the Best Way to Cluster NCI-60 Molecules. Biomolecules.

[42] Illing E.A., Cho D.-Y., Zhang S., Skinner D.F., Dunlap Q.A., Sorscher E.J., Woodworth B.A. (2015). Chlorogenic Acid Activates CFTR-Mediated Cl- Secretion in Mice and Humans: Therapeutic Implications for Chronic Rhinosinusitis. Otolaryngol. Head. Neck Surg..

[43] GULCIN I. (2006). Antioxidant Activity of Caffeic Acid (3,4-Dihydroxycinnamic Acid). Toxicology.

[44] Oboh G., Agunloye O.M., Adefegha S.A., Akinyemi A.J., Ademiluyi A.O. (2015). Caffeic and Chlorogenic Acids Inhibit Key Enzymes Linked to Type 2 Diabetes (in Vitro): A Comparative Study. J. Basic Clin. Physiol. Pharmacol..

[45] Giresha A.S., Urs D., Pundalik S., Meti R.S., Pramod S.N., Supreetha B.H., Somegowda M., Dharmappa K.K., El-Shehawi A.M., Albogami S. (2022). Sinapicacid Inhibits Group IIA Secretory Phospholipase A2 and Its Inflammatory Response in Mice. Antioxidants.

[46] Supuran C.T. (2008). Carbonic Anhydrases: Novel Therapeutic Applications for Inhibitors and Activators. Nat. Rev. Drug Discov..

[47] Temperini C., Innocenti A., Scozzafava A., Mastrolorenzo A., Supuran C.T. (2007). Carbonic Anhydrase Activators: L-Adrenaline Plugs the Active Site Entrance of Isozyme II, Activating Better Isoforms I, IV, VA, VII, and XIV. Bioorg. Med. Chem. Lett..

[48] Lomelino C.L., McKenna R. (2019). Carbonic Anhydrase II in Complex with Carboxylic Acid-Based Inhibitors. Acta Crystallogr. Sect. F Struct. Biol. Commun..

[49] Alterio V., Monti S.M., Truppo E., Pedone C., Supuran C.T., De Simone G. (2010). The First Example of a Significant Active Site Conformational Rearrangement in a Carbonic Anhydrase-Inhibitor Adduct: The Carbonic Anhydrase I–Topiramate Complex. Org. Biomol. Chem..

[50] Nocentini A., Alterio V., Bua S., Micheli L., Esposito D., Buonanno M., Bartolucci G., Osman S.M., ALOthman Z.A., Cirilli R. (2020). Phenyl(Thio)Phosphon(amid)Ate Benzenesulfonamides as Potent and Selective Inhibitors of Human Carbonic Anhydrases II and VII Counteract Allodynia in a Mouse Model of Oxaliplatin-Induced Neuropathy. J. Med. Chem..

[51] Waheed A., Sly W.S. (2017). Carbonic Anhydrase XII Functions in Health and Disease. Gene.

[52] Alterio V., Pan P., Parkkila S., Buonanno M., Supuran C.T., Monti S.M., De Simone G. (2014). The Structural Comparison between Membrane-associated Human Carbonic Anhydrases Provides Insights into Drug Design of Selective Inhibitors. Biopolymers.

[53] Duda D.M., Tu C., Fisher S.Z., An H., Yoshioka C., Govindasamy L., Laipis P.J., Agbandje-McKenna M., Silverman D.N., McKenna R. (2005). Human Carbonic Anhydrase III: Structural and Kinetic Study of Catalysis and Proton Transfer. Biochemistry.

[54] Mickevičiūtė A., Timm D.D., Gedgaudas M., Linkuvienė V., Chen Z., Waheed A., Michailovienė V., Zubrienė A., Smirnov A., Čapkauskaitė E. (2018). Intrinsic Thermodynamics of High Affinity Inhibitor Binding to Recombinant Human Carbonic Anhydrase IV. Eur. Biophys. J..

[55] Kazokaitė J., Kairys V., Smirnovienė J., Smirnov A., Manakova E., Tolvanen M., Parkkila S., Matulis D. (2019). Engineered Carbonic Anhydrase VI-Mimic Enzyme Switched the Structure and Affinities of Inhibitors. Sci. Rep..

[56] Zakšauskas A., Čapkauskaitė E., Paketurytė-Latvė V., Smirnov A., Leitans J., Kazaks A., Dvinskis E., Stančaitis L., Mickevičiūtė A., Jachno J. (2021). Methyl 2-Halo-4-Substituted-5-Sulfamoyl-Benzoates as High Affinity and Selective Inhibitors of Carbonic Anhydrase IX. Int. J. Mol. Sci..

[57] Di Fiore A., Monti S.M., Hilvo M., Parkkila S., Romano V., Scaloni A., Pedone C., Scozzafava A., Supuran C.T., De Simone G. (2009). Crystal Structure of Human Carbonic Anhydrase XIII and Its Complex with the Inhibitor Acetazolamide. Proteins Struct. Funct. Bioinforma..

[58] Lee S.-K., Boron W.F., Occhipinti R. (2023). Potential Novel Role of Membrane-Associated Carbonic Anhydrases in the Kidney. Int. J. Mol. Sci..

[59] Figueredo K.C., Guex C.G., Graiczik J., Reginato F.Z., Engelmann A.M., De Andrade C.M., Timmers L.F.S.M., Bauermann L.D.F. (2022). Caffeic Acid and Ferulic Acid Can Improve Toxicological Damage Caused by Iron Overload Mediated by Carbonic Anhydrase Inhibition. Drug Chem. Toxicol..

[60] Gülçin İ., Scozzafava A., Supuran C.T., Akıncıoğlu H., Koksal Z., Turkan F., Alwasel S. (2016). The Effect of Caffeic Acid Phenethyl Ester (CAPE) on Metabolic Enzymes Including Acetylcholinesterase, Butyrylcholinesterase, Glutathione S-Transferase, Lactoperoxidase, and Carbonic Anhydrase Isoenzymes I, II, IX, and XII. J. Enzyme Inhib. Med. Chem..

[61] Karpuzoglu E., Holladay S.D., Gogal R.M. (2013). Parabens: Potential Impact of Low-Affinity Estrogen Receptor Binding Chemicals on Human Health. J. Toxicol. Environ. Heal. Part B.

[62] Kciuk M., Gielecińska A., Mujwar S., Mojzych M., Marciniak B., Drozda R., Kontek R. (2022). Targeting Carbonic Anhydrase IX and XII Isoforms with Small Molecule Inhibitors and Monoclonal Antibodies. J. Enzyme Inhib. Med. Chem..

[63] Zou H., Ye H., Zhang J., Ren L. (2022). Recent Advances in Nuclear Receptors-Mediated Health Benefits of Blueberry. Phytomedicine.

[64] Taheri Y., Suleria H.A.R., Martins N., Sytar O., Beyatli A., Yeskaliyeva B., Seitimova G., Salehi B., Semwal P., Painuli S. (2020). Myricetin Bioactive Effects: Moving from Preclinical Evidence to Potential Clinical Applications. BMC Complement. Med. Ther..

[65] Mishra C.B., Tiwari M., Supuran C.T. (2020). Progress in the Development of Human Carbonic Anhydrase Inhibitors and Their Pharmacological Applications: Where Are We Today?. Med. Res. Rev..

[66] Kelly E., Vyas P., Weber J.T. (2017). Biochemical Properties and Neuroprotective Effects of Compounds in Various Species of Berries. Molecules.

[67] Ciccone L., Cerri C., Nencetti S., Orlandini E. (2021). Carbonic Anhydrase Inhibitors and Epilepsy: State of the Art and Future Perspectives. Molecules.

[68] García-Llorca A., Carta F., Supuran C.T., Eysteinsson T. (2024). Carbonic Anhydrase, Its Inhibitors and Vascular Function. Front. Mol. Biosci..

[69] Sobolev A.P., Ciampa A., Ingallina C., Mannina L., Capitani D., Ernesti I., Maggi E., Businaro R., Del Ben M., Engel P. (2019). Blueberry-Based Meals for Obese Patients with Metabolic Syndrome: A Multidisciplinary Metabolomic Pilot Study. Metabolites.

[70] Chan S.M.H., Selemidis S., Bozinovski S., Vlahos R. (2019). Pathobiological Mechanisms Underlying Metabolic Syndrome (MetS) in Chronic Obstructive Pulmonary Disease (COPD): Clinical Significance and Therapeutic Strategies. Pharmacol. Ther..

[71] de Oliveira M.S., Pellenz F.M., de Souza B.M., Crispim D. (2022). Blueberry Consumption and Changes in Obesity and Diabetes Mellitus Outcomes: A Systematic Review. Metabolites.

[72] Daina A., Michielin O., Zoete V. (2017). SwissADME: A Free Web Tool to Evaluate Pharmacokinetics, Drug-Likeness and Medicinal Chemistry Friendliness of Small Molecules. Sci. Rep..

[73] Djoumbou-Feunang Y., Fiamoncini J., Gil-de-la-Fuente A., Greiner R., Manach C., Wishart D.S. (2019). BioTransformer: A Comprehensive Computational Tool for Small Molecule Metabolism Prediction and Metabolite Identification. J. Cheminform..

