# Supplementary material for: Unraveling Protein-Metabolite Interactions in Precision Nutrition: A Case Study of Blueberry-Derived Metabolites Using Advanced Computational Methods
Source: Metabolites. 2024 Aug 3;14(8):430. doi: 10.3390/metabo14080430 (PMC11356322; doi:10.3390/metabo14080430)
Supplement: Supplementary file 1 [file metabolites-14-00430-s001.zip › Supplementary Table S1.pdf]

Supplementary Table S1: Protein targets for Cluster-0 metabolites predicted from SWISS target prediction. The number of identified protein targets increases progressively when the confidence score threshold is relaxed, allowing for hits with confidence levels 60-90%.

Cluster-0 metabolites	Protein target (partial hit, > 60% confidence hit)
4-Hydroxybenzoic acid	Alpha-(1,3)-fucosyltransferase 7, Steroid 5-alpha-reductase 2, Aldo-keto reductase family 1 member C1, C2
3,4-Dihydroxyhydrocinnamic acid	Tyrosine-protein kinase FYN, Tyrosine-protein kinase LCK, Epidermal growth factor receptor erbB1
3-(4-Hydroxyphenyl) propionic acid (Desaminotyrosine)	Estrogen receptor beta, Tyrosine-protein kinase FYN, Tyrosine-protein kinase LCK, Epidermal growth factor receptor erbB1
3,4-Dihydroxybenzeneacetic acid	14-3-3 protein gamma, Aldose reductase, Carbonic anhydrase II, Matrix metalloproteinase 9, Matrix metalloproteinase 1, Matrix metalloproteinase 2, G protein-coupled receptor 44
2-Benzamidoacetic acid (Hippuric acid)	Aldose reductase (by homology), Solute carrier family 22 member 6, Carbonic anhydrase XII, Carbonic anhydrase IX
3,5-Dihydroxybenzoic acid	Carbonic anhydrase VI, Carbonic anhydrase IV, Carbonic anhydrase III, Carbonic anhydrase VB, Carbonic anhydrase VA, Carbonic anhydrase XIII
3,4-Dihydroxybenzoic acid (Protocatechuic acid)	Carbonic anhydrase III, Carbonic anhydrase VB, Carbonic anhydrase VA, Carbonic anhydrase XIII
2,4-Dihydroxybenzoic acid	Carbonic anhydrase IX, Carbonic anhydrase VII, Carbonic anhydrase XIV, Carbonic anhydrase VI, Carbonic anhydrase IV, Carbonic anhydrase III, Carbonic anhydrase VB, Carbonic anhydrase VA, Carbonic anhydrase XIII
4-Hydroxycinnamic acid	Macrophage migration inhibitory factor, Arachidonate 5-lipoxygenase, Matrix metalloproteinase 9, Matrix metalloproteinase 1
2,3 –Dihydroxybenzoic acid (2-Pyrocatechuic acid)	Carbonic anhydrase II, Carbonic anhydrase I, Carbonic anhydrase XII, Carbonic anhydrase IX, Carbonic anhydrase VI, Carbonic anhydrase VII, Carbonic anhydrase XIV, Carbonic anhydrase IV
2,4,6-Trihydroxybenzaldehyde	Serine/threonine-protein kinase/endoribonuclease IRE1, Carbonic anhydrase II, Carbonic anhydrase I, Carbonic anhydrase XII, Carbonic anhydrase VII, Carbonic anhydrase XIV, Carbonic anhydrase IX
2-Hydroxybenzoic acid (Salicylic acid)	Carbonic anhydrase IX, Carbonic anhydrase VII, Carbonic anhydrase XIV, Carbonic anhydrase VI, Carbonic anhydrase III, Carbonic anhydrase IV, Carbonic anhydrase VB, Carbonic anhydrase VA, Carbonic anhydrase XIII
3-Hydroxybenzoic acid	Carbonic anhydrase VII, Carbonic anhydrase XIV, Carbonic anhydrase IV, Carbonic anhydrase III, Carbonic anhydrase VB, Carbonic anhydrase VA, Carbonic anhydrase XIII
3-Hydroxyhippuric acid	Aldose reductase (by homology), Carbonic anhydrase XII, Carbonic anhydrase IX, Solute carrier family 13 member 5, AMP deaminase 3
4-Hydroxybenzyl alcohol	Carbonic anhydrase II, Acetylcholinesterase, Carbonic anhydrase III, Carbonic anhydrase VI, Carbonic anhydrase XII, Carbonic anhydrase XIV, Carbonic anhydrase IX, Carbonic anhydrase IV
