# Supplementary material for: Unraveling Protein-Metabolite Interactions in Precision Nutrition: A Case Study of Blueberry-Derived Metabolites Using Advanced Computational Methods
Source: Metabolites. 2024 Aug 3;14(8):430. doi: 10.3390/metabo14080430 (PMC11356322; doi:10.3390/metabo14080430)
Supplement: Supplementary file 1 [file metabolites-14-00430-s001.zip › Supplementary Table S2.pdf]

Supplementary Table S2: Protein targets for Cluster-1 metabolites predicted from SWISS target prediction. Similar to cluster-0, when the confidence score threshold is relaxed to include partial hits with a confidence score of 60-90%, the number of predicted protein targets increases across diverse categories.

Cluster-1 metabolites	Protein target (partial hit, >60% confidence hit)
4-Hydroxy-3-methoxyphenylacetic acid (Homovanillic acid)	Cyclooxygenase-1, Cyclooxygenase-2, Aldose reductase, 14-3-3 protein gamma, Aldo-keto-reductase family 1 member C3, Carbonic anhydrase II, G protein-coupled receptor 44, Solute carrier family 13 member 5, Carboxypeptidase A1, Kynurenine 3-monooxygenase (by homology), Fructose-1,6-bisphosphatase, Lysine-specific demethylase 4A
4-Hydroxy-3,5-dimethoxybenzoic acid (Syringic acid)	-
Chlorogenic acid (3-Caffeoylquinic acid)	Aldose reductase, Aldo-keto reductase family 1 member B10, Matrix metalloproteinase 13, Matrix metalloproteinase 2, Beta amyloid A4 protein, Matrix metalloproteinase 12, Glucose-6-phosphate translocase, Carbonic anhydrase II, Carbonic anhydrase I, Carbonic anhydrase XII, Carbonic anhydrase IX, Liver glycogen phosphorylase, Protein kinase C delta (by homology), Protein kinase C alpha, Sialidase 4, Beta-secretase 1, Caspase-3
3-Methoxybenzenepropanoic acid	Cyclooxygenase-2, Thromboxane-A synthase, Egl nine homolog 3, Prostanoid FP receptor, C-terminal-binding protein 2, Serotonin 1a (5-HT1a) receptor, Prostanoid DP receptor, Lysine-specific demethylase 2A, Kynurenine 3-monooxygenase (by homology), Norepinephrine transporter, Cytochrome P450 1A2
3-(3-hydroxy-4-methoxyphenyl)propanoic acid	Cyclooxygenase-2, Thromboxane-A synthase, Egl nine homolog 3, Prostanoid FP receptor, C-terminal-binding protein 2, Serotonin 1a (5-HT1a) receptor, Prostanoid DP receptor, Lysine-specific demethylase 2A
4-Hydroxy-3-methoxycinnamic acid (Ferulic acid)	Carbonic anhydrase VB, Arachidonate 5-lipoxygenase, Matrix metalloproteinase 9, Matrix metalloproteinase 1, Matrix metalloproteinase 2, Protein-tyrosine phosphatase 1B, Monoamine oxidase B, Carbonic anhydrase XIII (by homology), Aldose reductase, Carbonic anhydrase III, Beta amyloid A4 protein, Nuclear factor erythroid 2-related factor 2
3,5-Dihydroxy-4-methoxybenzoic acid (4-O-Methylgallic acid)	Carbonic anhydrase II, Carbonic anhydrase VII, Carbonic anhydrase I, Carbonic anhydrase III, Carbonic anhydrase VI, Carbonic anhydrase XII, Carbonic anhydrase XIV, Carbonic anhydrase IX, Carbonic anhydrase VA, Alpha-(1,3)-fucosyltransferase 7
3-(4-Hydroxy-3-methoxyphenyl) propanoic acid (Hydroferulic acid)	Tyrosine-protein kinase FYN, Tyrosine-protein kinase LCK, Testis-specific androgen-binding protein, Epidermal growth factor receptor erbB1, Dual specificity mitogen-activated protein kinase kinase 1
3-Hydroxy-4-methoxybenzoic acid	Carbonic anhydrase II, Carbonic anhydrase I, Carbonic anhydrase XII, Carbonic anhydrase IX, Thiopurine S-methyltransferase, Carbonic anhydrase VII, Carbonic Anhydrase VA
4-Hydroxy-3,5-dimethoxycinnamic acid (Sinapic acid)	Carbonic anhydrase II, Carbonic anhydrase VII, Carbonic anhydrase I, Carbonic anhydrase VI, Carbonic anhydrase XII, Carbonic anhydrase XIV, Carbonic anhydrase IX, Carbonic anhydrase VA, Carbonic anhydrase VB, Protein-tyrosine phosphatase 1B, Aldose reductase, Tubulin beta-1 chain, Tubulin beta-3 chain, Quinone reductase 2, Cytochrome P450 1B1,
4-Hydroxy-3-methoxybenzoic acid (Vanillic acid)	Carbonic anhydrase II, Carbonic anhydrase VII, Carbonic anhydrase I, Carbonic anhydrase XII, Carbonic anhydrase XIV, Carbonic anhydrase IX, Carbonic anhydrase VI, Carbonic anhydrase III, Carbonic anhydrase VA, Carbonic anhydrase IV, Thiopurine S-methyltransferase, DNA polymerase alpha subunit, DNA polymerase beta, Plasminogen activator inhibitor-1, Tubulin beta-1 chain
4-Hydroxybenzoic acid methyl ester (Methylparaben)	Carbonic anhydrase II, Carbonic anhydrase VII, Carbonic anhydrase I, Carbonic anhydrase XII, Carbonic anhydrase XIV, Carbonic anhydrase IX, Carbonic anhydrase III, Carbonic anhydrase VI, Carbonic anhydrase VA, Carbonic anhydrase IV, Thiopurine S-methyltransferase, Transthyretin, Carbonic anhydrase VB, Carbonic anhydrase XIII (by homology),
