# Supplementary material for: Unraveling Protein-Metabolite Interactions in Precision Nutrition: A Case Study of Blueberry-Derived Metabolites Using Advanced Computational Methods
Source: Metabolites. 2024 Aug 3;14(8):430. doi: 10.3390/metabo14080430 (PMC11356322; doi:10.3390/metabo14080430)
Supplement: Supplementary file 1 [file metabolites-14-00430-s001.zip › Supplementary Table S3.pdf]

Table S3: Docking scores of Cluster-0 metabolites with four different proteins. The predicted binding energies for many Cluster-0 metabolites indicate strong binding, although a few displayed lower binding energies. Docking energy is expressed in *kcal/mol*.

**Cluster-0 metabolites**	**D(1A) Dopamine Receptor**	**Epidermal Growth Factor Receptor**	**Estrogen Receptor Beta**	**Macrophage Migration Inhibitory Factor**
2,4,6-trihydroxybenzaldehyde	-6.2	-5.1	-5.9	-4.8
2,4-Dihydroxybenzoic acid	-6.3	-5.7	-6.2	-5.3
2,3-Dihydroxybenzoic acid (2-Pyrocatechuic acid)	-6.6	-5.7	-6.1	-5.4
3,4-Dihydroxybenzeneacetic acid	-6.4	-5.8	-6.2	-5.7
3,4-Dihydroxyhydrocinnamic acid	-6.6	-6.1	-6.6	-5.9
3,5-Dihydroxybenzoic acid	-6.3	-5.4	-6.1	-5.2
3-Hydroxybenzoic acid	-6.4	-5.3	-5.9	-5.3
3-Hydroxyhippuric acid	-6.3	-6.2	-7.2	-6.4
4-Hydroxybenzoic acid	-6.2	-5.4	-5.9	-5.4
4-Hydroxybenzyl alcohol	-5.6	-4.7	-5.4	-4.9
4-Hydroxycinnamic acid	-6.3	-5.8	-6.2	-5.9
3-(4-Hydroxyphenyl) propionic acid (Desaminotyrosine)	-6.0	-5.8	-6.3	-5.8
2-Benzamidoacetic acid (Hippuric acid)	-6.5	-5.7	-6.9	-6.5
3,4-Dihydroxybenzoic acid (Protocatechuic acid)	-6.5	-5.6	-6.1	-5.5
2-Hydroxybenzoic acid (Salicylic acid)	-6.4	-5.6	-6.1	-5.4
