# Supplementary figures and images for: Unraveling Protein-Metabolite Interactions in Precision Nutrition: A Case Study of Blueberry-Derived Metabolites Using Advanced Computational Methods
Source: Metabolites. 2024 Aug 3;14(8):430. doi: 10.3390/metabo14080430 (PMC11356322; doi:10.3390/metabo14080430)

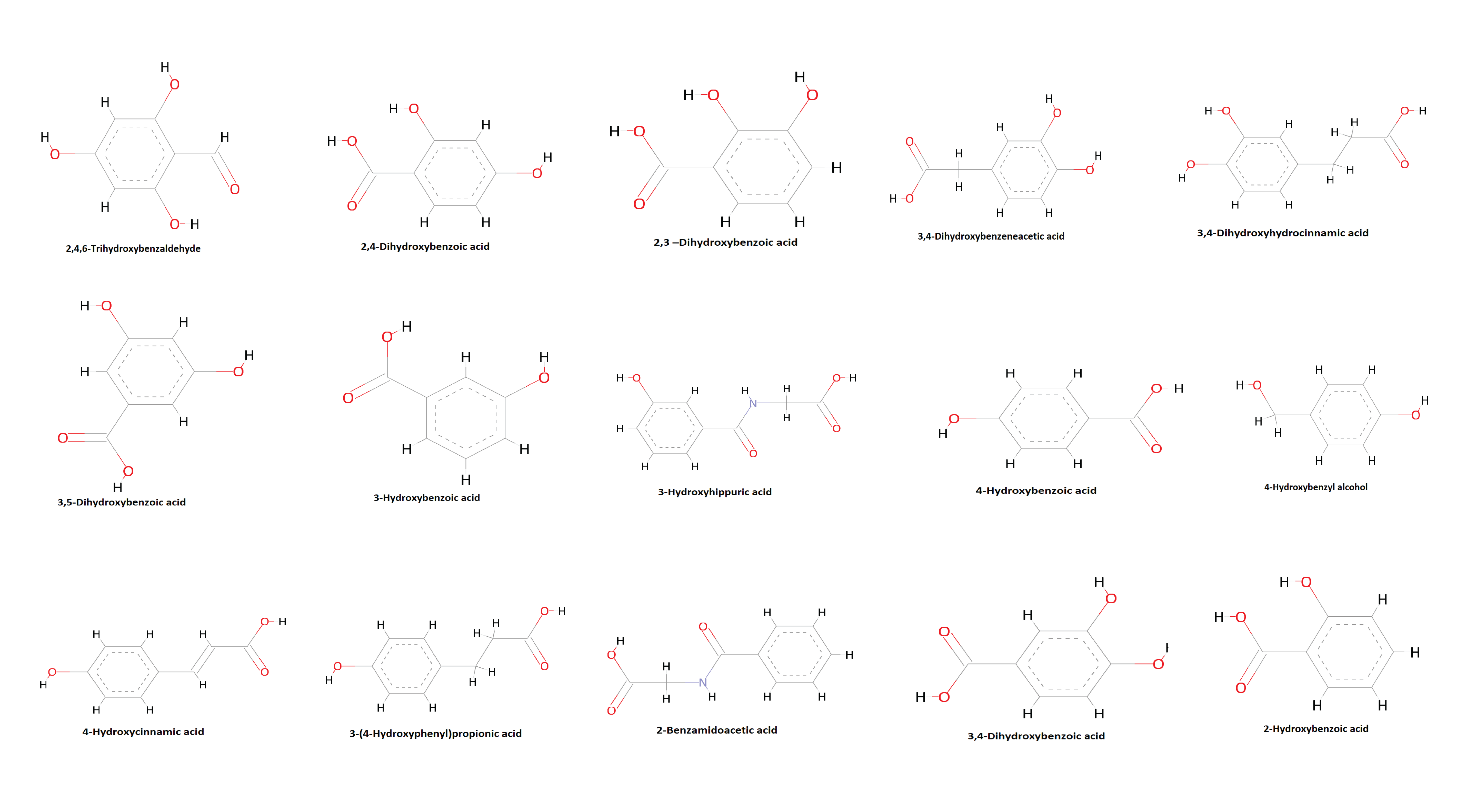

Supplement: Supplementary file 1 [file metabolites-14-00430-s001.zip › Supplementary Figure S1.tif]

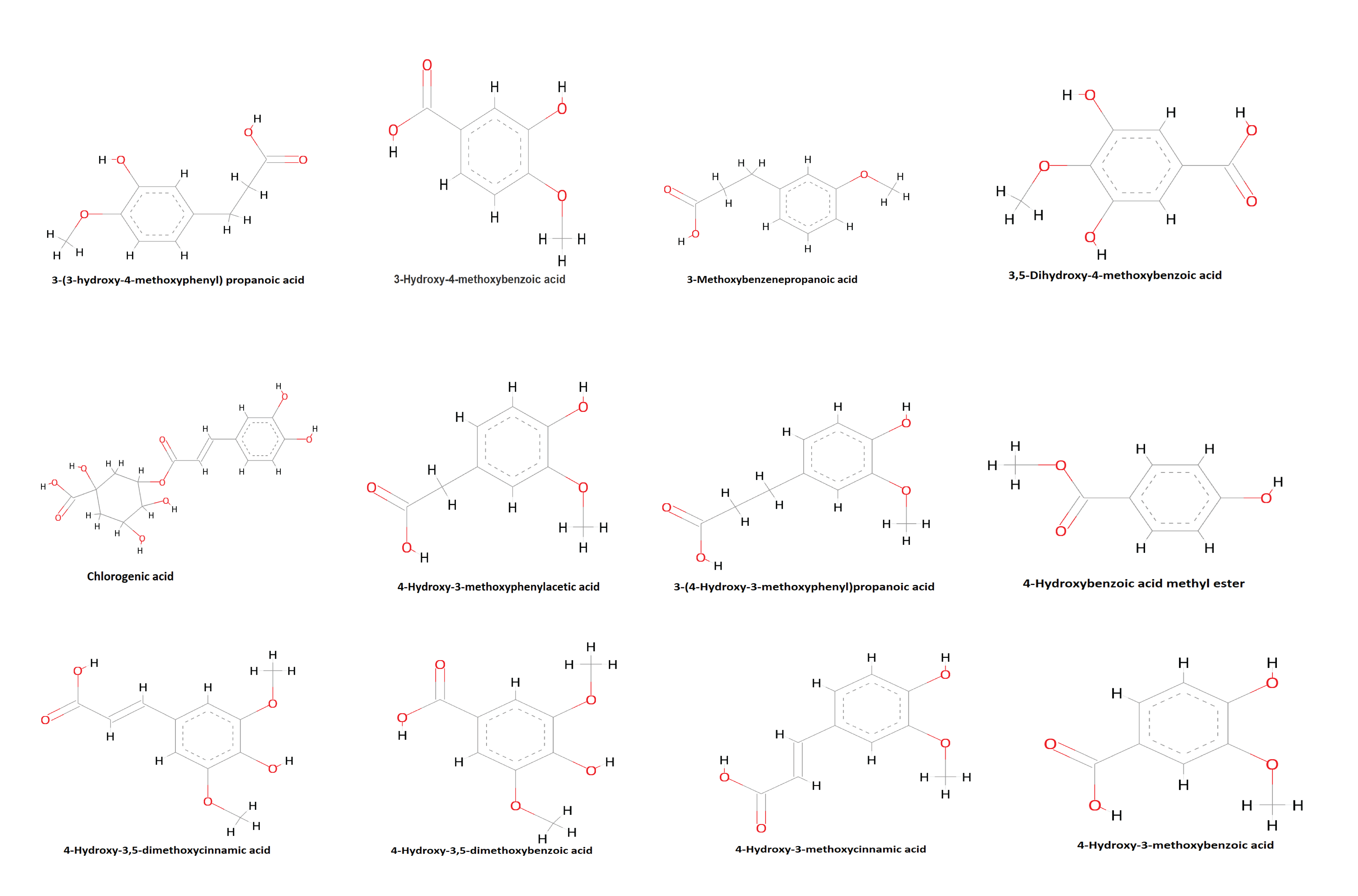

Supplement: Supplementary file 1 [file metabolites-14-00430-s001.zip › Supplementary Figure S2.tif]

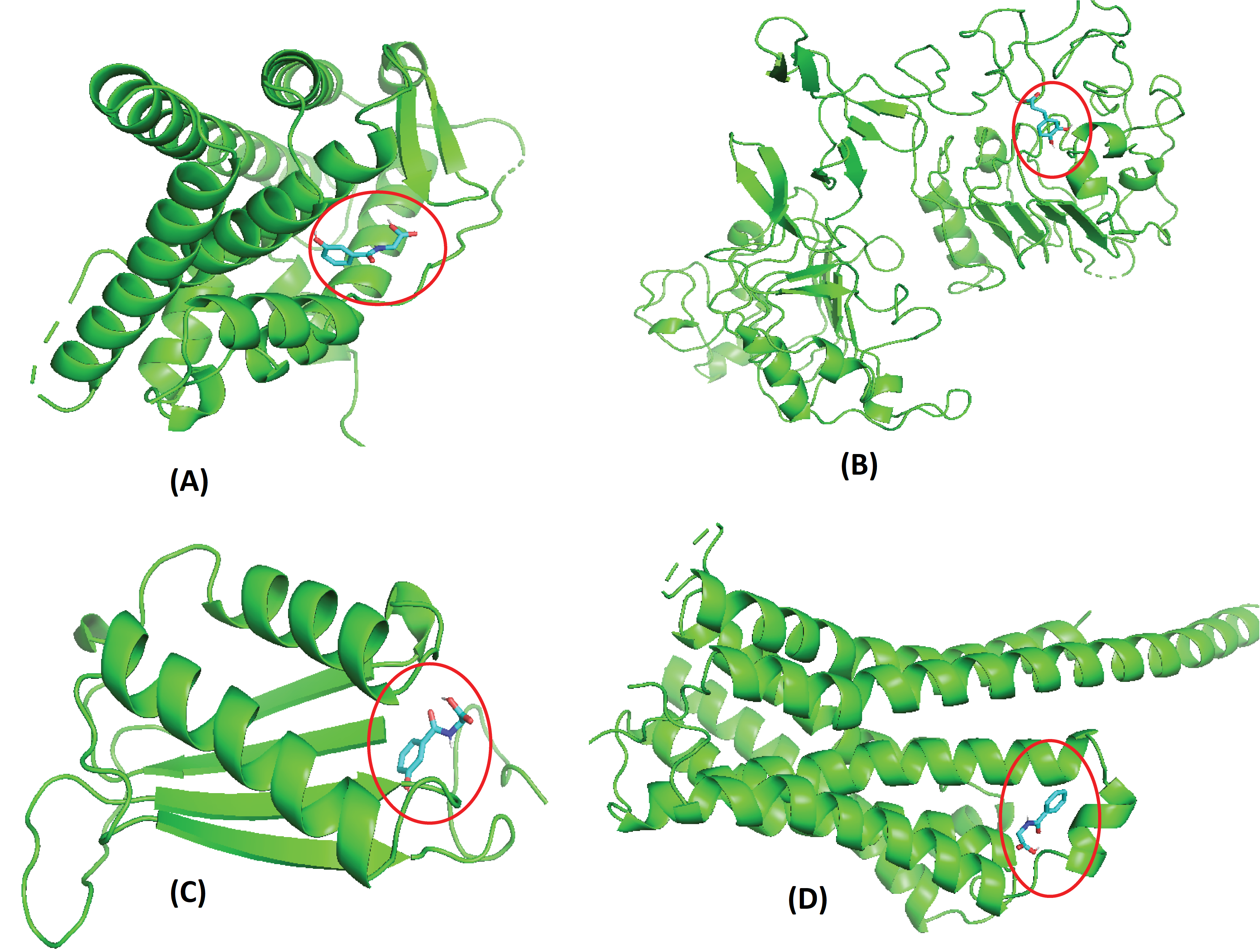

Supplement: Supplementary file 1 [file metabolites-14-00430-s001.zip › Supplementary Figure S3.tif]

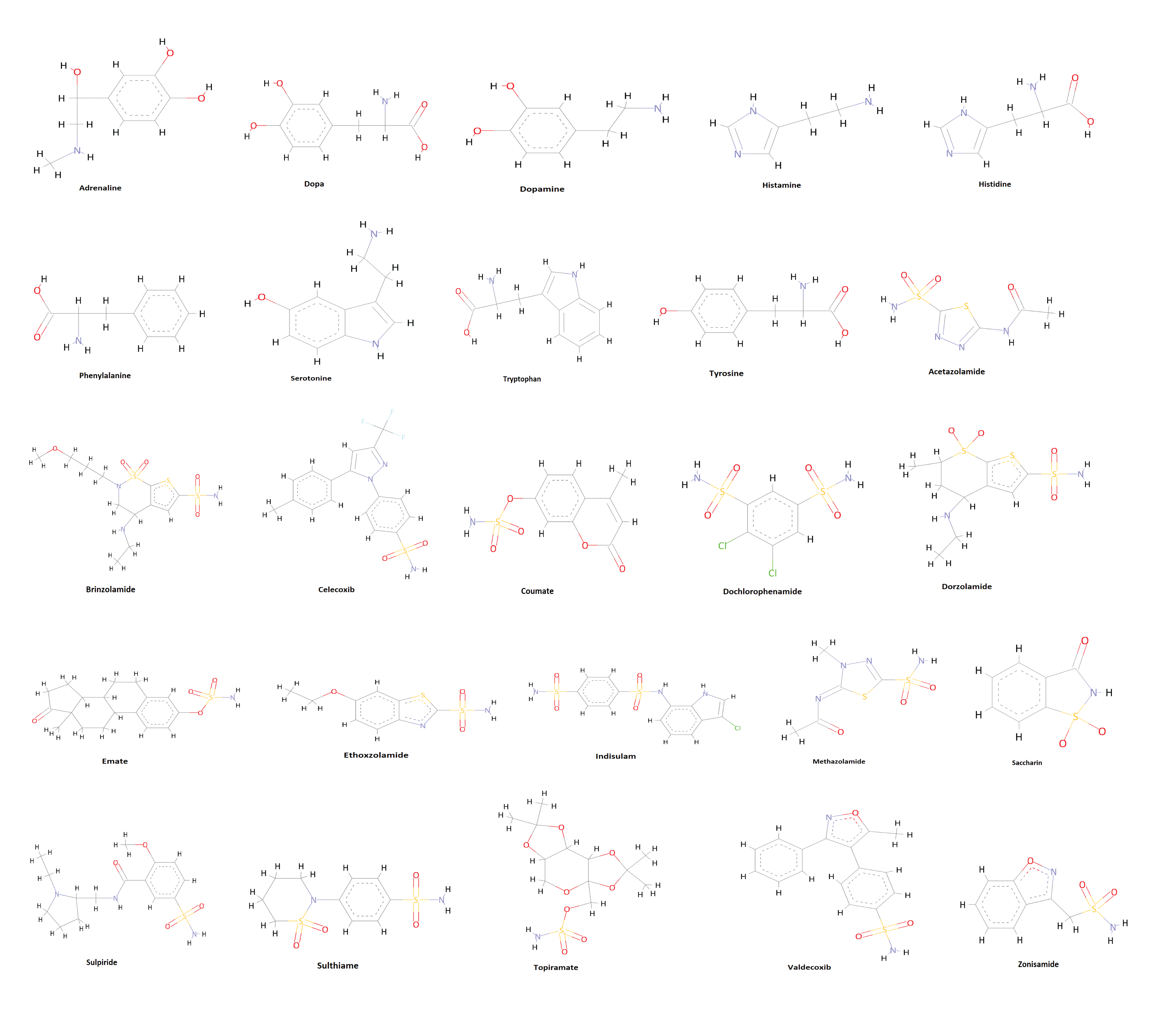

Supplement: Supplementary file 1 [file metabolites-14-00430-s001.zip › Supplementary Figure S4.tif]

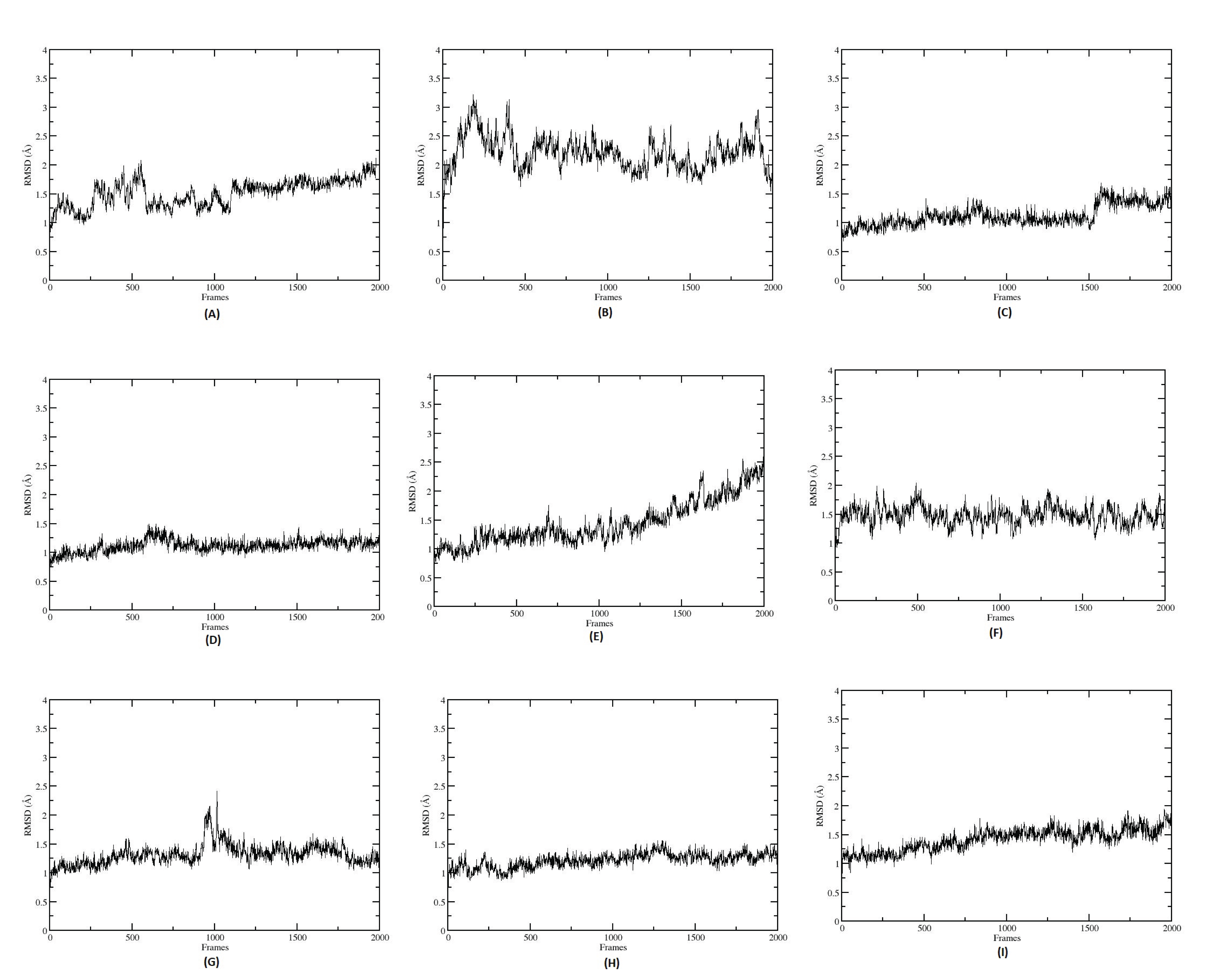

Supplement: Supplementary file 1 [file metabolites-14-00430-s001.zip › Supplementary Figure S5.tif]

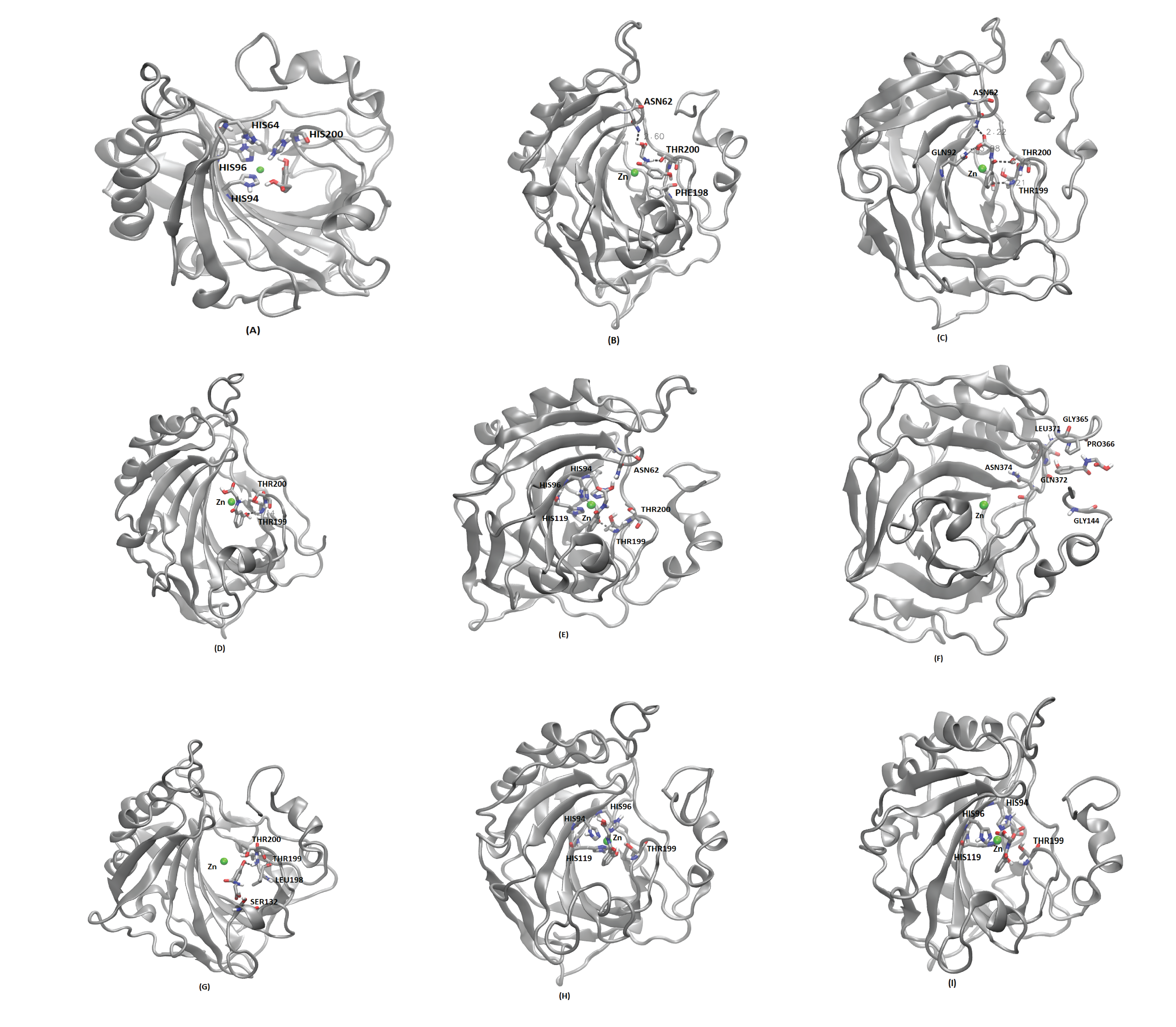

Supplement: Supplementary file 1 [file metabolites-14-00430-s001.zip › Supplementary Figure S6.tif]
